# NO-cGMP Signaling in Endothelial Function of the Healthy and Inflamed Dental Pulp

**DOI:** 10.3390/ijms27010057

**Published:** 2025-12-20

**Authors:** Yüksel Korkmaz, Tobias Kollmar, Judith F. Schultheis, Pablo Cores Ziskoven, Lena K. Müller-Heupt, James Deschner

**Affiliations:** Department of Periodontology and Operative Dentistry, University Medical Center, Johannes Gutenberg University Mainz, 55131 Mainz, Germany

**Keywords:** nitric oxide (NO), endothelial nitric oxide synthase (eNOS), NO-sensitive guanylyl cyclase (NO-GC), cyclic guanosine 3′,5′-monophosphate (cGMP), healthy dental pulp, caries, inflamed dental pulp, reactive oxygen species (ROS), reactive nitrogen species (RNS), endothelial dysfunction

## Abstract

The intra- and intercellular signaling molecule nitric oxide (NO) is produced in endothelial cells by the activity of endothelial NO synthase (eNOS). Upon formation, NO diffuses into the underlying vascular smooth muscle cells, where it activates NO-sensitive guanylyl cyclase (NO-GC) resulting in the production of cyclic guanosine 3′,5′-monophosphate (cGMP) from guanosine 5′-triphosphate (GTP). Inducing vasodilatation, inhibiting platelet aggregation and leukocyte adhesion, and inhibiting the proliferation and migration of vascular smooth muscle cells, the NO-cGMP signaling leads to a number of anti-inflammatory processes. Inflammation-dependent elevated concentrations of reactive oxygen species (ROS) and reactive nitrogen species (RNS) in blood vessels of inflamed dental pulp induce an uncoupling of eNOS and oxidized NO-GC, leading to a disruption of NO-cGMP signaling. Endothelial dysfunction in inflamed dental pulp alters cell–cell and cell–matrix interactions, reducing the regenerative and reparative potential of the dentin–pulp complex in response to carious lesions. In the therapeutic management of caries, it is essential to consider the presence of endothelial dysfunction in the inflamed dental pulp. The utilization of NO-GC stimulators and activators in indirect and direct pulp capping materials may enhance the regeneration and repair potential of inflamed dental pulp.

## 1. Introduction

The dental pulp and dentin form a structural and functional unit called the dentin–pulp complex. The dental pulp is supplied via the terminal arteries and veins through the apical foramen [1,2,3]. The microcirculation of the dental pulp is responsible for supplying the cells with oxygen, nutrients, and ions, as well as for removing metabolic waste products from the cells of the dentin–pulp complex. In cell–cell and cell–matrix interactions, the cells of the dental pulp perform a variety of complex biological functions to maintain homeostasis in the healthy state of the dentin–pulp complex. In the case of inflammation induced by caries, the cells of the dental pulp have the capacity to regulate the process of regeneration and repair in the dentin–pulp complex [4,5,6,7]. Thus, the microcirculation in the dentin–pulp complex plays a crucial role in maintaining homeostasis under physiological conditions, as well as in regenerating and repairing the tissue under inflammatory conditions.

Under physiological conditions, the endothelium is adapted to the unique structure of the dental pulp and is able to respond appropriately to tissue-specific signals according to the needs and functions of the dentin–pulp complex. As the dental pulp is located within a rigid, mineralized extracellular dentin matrix with limited capacity to expand in response to caries-induced inflammation [8,9,10,11], this unique structure, which is not found in other organs, highlights the critical role of endothelial function in the dental pulp during inflammation [12].

In the blood vessels, endothelial cells maintain homeostasis by producing and releasing nitric oxide (NO), prostaglandins, and endothelin [13,14,15]. Upon activation by NO at physiological concentrations [16], NO-sensitive guanylyl cyclase (NO-GC) induces the production of cyclic guanosine 3′,5′-monophosphate (cGMP) from guanosine 5′-triphosphate (GTP) in vascular smooth muscle cells (VSMCs) [17,18]. The NO-cGMP signaling is implicated in a variety of cell functions in a cell-specific manner, including the induction of vasodilation [18,19], the inhibition of platelet aggregation [20,21,22,23] and leukocyte adhesion [24,25,26,27], and the inhibition of the proliferation and migration of VSMCs [28,29,30,31].

Studies on the cardiovascular system have reported the potential benefits of drugs that restore or improve the impaired and altered functions of the NO-cGMP signaling cascade in blood vessels under inflammatory conditions [32,33,34,35,36,37]. Here, we provide a comparative review of the current state of knowledge regarding NO-cGMP signaling in endothelial function and dysfunction in the cardiovascular system, as well as in the blood vessels of healthy and inflamed dental pulp. Finally, we propose the therapeutic potential of NO-cGMP signaling for treating endothelial dysfunction in inflamed dental pulp in response to carious lesions.

## 2. NO and NO-cGMP Signaling in Endothelial Function

The synthesis of cGMP is catalyzed by NO-GC [18,34] and natriuretic peptide (NP)-activated particulate guanylyl cyclase (pGC) [38,39]. However, the activation of NO-GC and pGC does not contribute to a common cGMP pool [40,41,42]. The production of cGMP through the activation of NO-GC and pGC is compartmentalized in different subcellular regions (e.g., by NO-GC in the cytosol and by pGC in membranes), resulting in different biological reactions [40,41,42]. For example, the absence of NP receptors in platelets has been described [21,22]. NO-GC is the only enzyme that catalyzes cGMP synthesis in platelets [21,22]. Thus, the formation of cGMP through the activation of NO-GC and pGC depends on cell type and subcellular compartmentalization of proteins expressed. In this review, NO-cGMP signaling is considered to elucidate endothelial function and dysfunction in blood vessels in both healthy and inflamed dental pulp.

### 2.1. The Structure, Expression, and Regulation of Nitric Oxide Synthases

**Figure 1:**
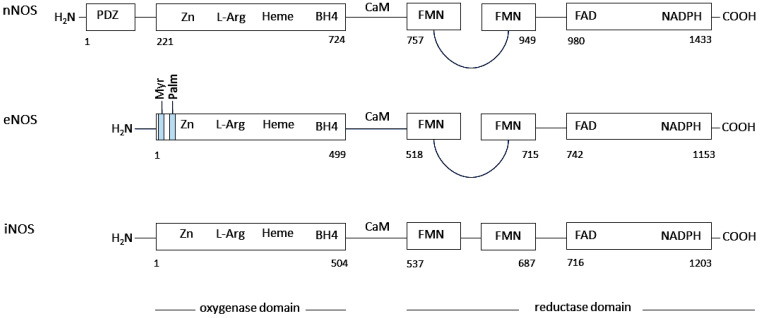
Protein domains of human nNOS, eNOS, and iNOS [45]. Human nNOS comprises 1434 amino acids and has a molecular weight of 161 kDa. Human eNOS contains 1153 amino acids and has a molecular weight of 131 kDa, while iNOS contains 1203 amino acids and has a molecular weight of 133 kDa. The amino acid residue numbers of nNOS, eNOS, and iNOS are indicated at the beginning and end of each domain. The enzyme nNOS contains PDZ, oxygenase, and reductase domains. The enzymes eNOS and iNOS contain oxygenase and reductase domains. The oxygenase and reductase domains are separated by a connecting region that contains a regulatory CaM binding domain. In their oxygenase domain, nNOS, eNOS, and iNOS contain binding sites for Zn, L-arginine, heme, and BH4. In the oxygenase domain, eNOS additionally contains the myristoylation (Myr) and palmitoylation sites (Palm). In their C-terminal reductase domain, nNOS, eNOS, and iNOS contain the binding sites for NADPH, flavin adenine dinucleotide (FAD), and flavin mononucleotide (FMN). The NH_2_-terminal domain of eNOS contains a glycine residue that is required for irreversible myristoylation of the enzyme. Myristoylation of the N-terminal glycine has been demonstrated to direct eNOS to the membrane [54], while reversible palmitoylation of Cys15 and Cys26 has been shown to specifically direct eNOS to caveolae [55,56].

The intra- and intercellular molecule NO is synthesized by NO-synthases (NOSs), which are encoded by separate genes. In different types of cells, NO is produced through the activity of neuronal (n), endothelial (e) and inducible (i) NOSs [43,44,45,46,47]. The structure of a NOS-monomer is composed of an N-terminal oxygenase and a C-terminal reductase domain, which are linked to a calmodulin (CaM) binding sequence. The N-terminal oxygenase domain of NOS contains binding sites for tetrahydrobiopterin (BH4) and iron protoporphyrin IX (heme). The C-terminal reductase domain of NOS contains nicotinamide adenine dinucleotide phosphate (NADPH)-, flavin adenine dinucleotide (FAD)- and flavin mononucleotide (FMN)-binding domains [46,47,48,49] (Figure 1).

In its monomeric form, NOS is unable to form NO. The synthesis of NO is catalyzed exclusively by NOSs in their dimeric form [48,50]. In the presence of dimeric form, electron transfer occurs from the reductase domain of one NOS-monomer to the oxygenase domain of the other NOS-monomer resulting in the formation of NO [32,51]. In order to form NO, NOSs utilize L-arginine and molecular oxygen (O_2_) as substrates. In this reaction, a NOS-monomer uses heme, BH4, FMN, FAD and NADPH as cofactors [46,47]. The reductase domain binds the cofactors FAD and FMN. NADPH acts as a source of electrons, which are then utilized by FAD. BH4 is essential for enzyme function and facilitates electron transfer. Heme is the only obligate cofactor for forming an active dimer of NOS [47,50]. It is also essential for transferring electrons between FMN domains in one monomer and heme in the other [32,47,49,50]. In the reductase domain of one monomer, the enzyme transfers electrons from NADPH to FAD, and from FAD to FMN. These electrons are then transferred to the heme iron in the oxygenase domain of the other monomer, reducing Fe^3+^ to Fe^2+^. O_2_ binds to Fe^2+^ and reacts with L-arginine to produce NO and L-citrulline [32,48,49,50,52].

Three different genes encode the three different isoforms of NOS: neuronal NOS (nNOS; NOSI) is encoded by the *NOS1* gene, endothelial NOS (eNOS; NOSIII) by the *NOS3* gene, and inducible NOS (iNOS; NOSII) by the *NOS2* gene [45,53]. The activities of nNOS and eNOS, which are constitutively expressed under physiological conditions, are regulated by transcriptional, post-transcriptional, and post-translational mechanisms (phosphorylation, acetylation, protein–protein interaction, S-nitrosylation, and S-glutathionylation) [43,47,53]. The activity of iNOS is mainly regulated by gene transcription under inflammatory conditions [47,53].

#### 2.1.1. Neuronal Nitric Oxide Synthase (nNOS)

The enzyme nNOS, encoded by the *NOS1* gene located at 12q24.2 on human chromosome 12, contains 1434 amino acids (a.a.) with a molecular mass of 160.8–161 kDa [45,53,57,58] (Figure 1). Under physiological conditions, nNOS is expressed in central [59,60], peripheral [61,62] neurons, in vascular smooth muscle cells [63,64] and in skeletal muscle fibers [65,66,67,68]. In these locations, nNOS regulates synaptic plasticity in the central nervous system [59,69,70,71,72,73,74], the relaxation of smooth [75] and skeletal muscles [66], and vasodilation via peripheral nitrergic nerves [76,77,78,79].

#### 2.1.2. Endothelial Nitric Oxide Synthase (eNOS)

The enzyme eNOS, encoded by the *NOS3* gene located at 7q35–7q36 on human chromosome 7, contains 1203 a.a. with a molecular mass of 133 kDa [43,45,53,80] (Figure 1). The eNOS enzyme is mainly expressed in endothelial cells [81,82,83,84]. Prior to the identification of NO in endothelial cells [81,82], it had been reported that endothelium-dependent relaxation could be mediated by the activity of NO-GC and cGMP-dependent protein phosphorylation [85,86,87]. Following the identification of the endothelium-dependent relaxation factor as NO [81,82,83,84], it was found that NO, formed in endothelial cells, activates its target enzyme NO-GC in VSMCs, which, in turn, induces increased formation of cGMP in VSMCs to regulate essential physiological functions in blood vessels via its downstream signalling cascade [17,18]. The synthesis of NO by the activity of eNOS in endothelial cells regulates the relaxation of VSMCs [19,47], mediates angiogenesis [53,88], inhibits VSMC proliferation [28,29,30,31], and inhibits platelet aggregation in the lumen of blood vessels [20,22]. In cardiovascular diseases, eNOS is uncoupled under inflammatory conditions, leading to endothelial dysfunction in blood vessels [32,37,89,90].

#### 2.1.3. Inducible Nitric Oxide Synthase (iNOS)

The enzyme iNOS, encoded by the *NOS2* gene located at 17q11.2-q12 on human chromosome 17, contains 1153 a.a. with a molecular mass of 130–131 kDa [45,53] (Figure 1). In contrast to eNOS and nNOS, iNOS activity is regulated independently of calcium [45,47,53,91]. The production of NO by iNOS activity depends primarily on the expression of iNOS, which is mainly regulated at the transcriptional level [92,93,94,95]. The activity of iNOS is transcriptionally regulated under inflammatory and oxidative conditions in a number of cells, including leukocytes, endothelial cells, VSMCs, cardiac muscle cells, nerve cells, and fibroblasts [53,96,97,98,99,100,101]. Once formed, the catalytic activity of dimeric iNOS is extremely high, and the enzyme maintains high NO production until the substrate and cofactors are depleted or the enzyme is degraded [53,91]. Therefore, the expression of iNOS, which is continuously regulated transcriptionally under inflammatory conditions, induces extremely increased NO formation in different types of cells and in blood vessel cells resulting in the formation of ROS and RNS and thus triggering pathological processes in the tissues.

### 2.2. The Regulation of eNOS Activity in Endothelium

**Figure 2:**
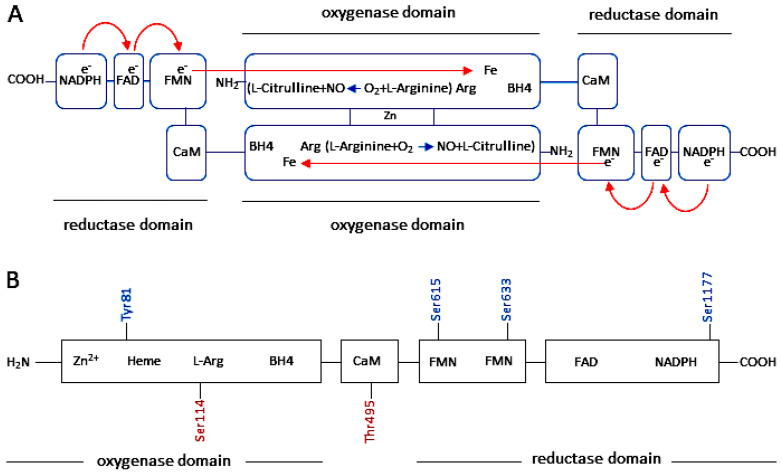
The synthesis of NO in endothelial cells by the eNOS dimer (coupled eNOS). (**A**) In the homodimeric form, eNOS oxidizes the substrate L-arginine to L-citrulline and NO. Each eNOS monomer is composed of an oxygenase domain and a reductase domain. The oxygenase domain is linked to the reductase domain via a calmodulin (CaM) binding sequence. The reductase domain contains cofactors nicotinamide adenine dinucleotide phosphate (NADPH)-, flavin adenine dinucleotide (FAD)- and flavin mononucleotide (FMN)-binding sequences. The oxygenase domain contains heme and tetrahydrobiopterin (BH4). Heme is an absolutely necessary cofactor for forming active eNOS dimers. Heme is also required for transferring electrons between the FMN domains and the heme of the opposite monomer. BH4 is essential for the functioning of the eNOS dimer, facilitating electron transfer. Electrons are transferred from NADPH via FAD and FMN of a monomer to heme iron of the other monomer, whereby Fe^3+^ is reduced to Fe^2+^. O_2_ then binds to Fe^2+^ and reacts with L-arginine to form NO and L-citrulline. (**B**) The activity of eNOS is mainly regulated post-translationally by phosphorylation of the enzyme at serine (Ser), threonine (Thr) and tyrosine (Tyr) residues. Phosphorylation of eNOS at Tyr81, Ser615, Ser633, and Ser1177 (in blue; in the human sequence) leads to increased enzyme activity, while phosphorylation of the enzyme at Ser114 and Thr495 (in red; in the human sequence) leads to a reduction in eNOS activation [47,48,102,108]. Phosphorylation of eNOS at Ser1177 increases electron flow through the reductase domain, thereby increasing the enzyme activity of eNOS. In contrast, the phosphorylation of eNOS at Thr495 leads to a decrease in electron flow, thus reducing the enzyme activity of eNOS [47,48]. The illustration was modified from [32,102].

The synthesis of eNOS occurs as monomers. The eNOS monomer is unable to bind the cofactor BH4 or the substrate L-arginine [47,50]. To produce NO, eNOS must be present in dimeric form. In dimeric form, it is referred to as “coupled eNOS” [47,48]. BH4 is an essential cofactor required for enzymatic eNOS activity. BH4 facilitates the transfer of electrons from NADPH in the reductase domain of one eNOS-monomer to the oxygenase domain of another eNOS-monomer, converting L-arginine into NO and L-citrulline through a reaction with O_2_ [47,48,49,50,102] (Figure 2).

In endothelial cells, eNOS is subcellularly localized in caveolae in the plasma membrane [102,103,104]. Under basal conditions, caveolin-1 maintains the inactive state of eNOS in endothelial cells [56,103]. This limits eNOS activation and production of NO [47,48,102]. In endothelial cells, stimulation by agonists (e.g., VEGF) increases intracellular calcium. This disrupts the interaction between caveolin-1 and eNOS via calcium-bound calmodulin [49,105,106]. Hsp90 then binds to eNOS, promoting the recruitment of Akt/PKB. In turn, Akt/PKB phosphorylates eNOS at Ser1177 [13,47,48,107]. The phosphatase calcineurin is also recruited to the vicinity of eNOS via binding to Hsp90. Calcineurin then dephosphorylates eNOS at Thr495, which contributes to maintaining NO release independently of the intracellular calcium level [47,48,49,106] (Figure 2).

### 2.3. Structure and Maturation of NO-GC

Upon stimulation by NO, NO-GC induces the production of cGMP from GTP. Therefore, as a receptor enzyme for NO, NO-GC plays a crucial role within the NO-cGMP signaling cascade [17,109,110,111].

#### 2.3.1. The Protein Structure of NO-GC

NO-GC is a heme-containing 150 kDa heterodimer protein composed of α (690 a.a. residues; 80 kDa) and β (620 a.a. residues; 70 kDa) subunits [112,113,114]. Each NO-GC subunit has two isoforms (α_1_/α_2_ and β_1_/β_2_) encoded by 4 separate genes [115,116]. The expression of the α_1_- and β_1_-subunits of NO-GC has been detected in various mammalian cell types [117,118,119,120], while the expression of the α_2_- [119,121,122,123] and β_2_- [124,125] subunits is tissue-specific. It has been established that enzymes containing the β_2_-subunit exhibit no enzymatic activity [126,127].

The α- and β-subunits of NO-GC are structured similarly in modular domains. The amino acid sequence of the α- and β-subunits was analyzed, identifying specific domains of NO-GC: an N-terminal H-NOX domain, a Per/Arnt/Sim (PAS) domain, a coiled-coil (CC) domain, and a C-terminal catalytic domain. In the N-terminal H-NOX domain, the β-subunit of NO-GC contains an iron-containing heme group to which NO binds [128,129,130,131]. The PAS and CC domains of NO-GC mediate protein–protein interactions. The catalytic domain is responsible for the enzymatic activity of NO-GC, in which GTP is catalyzed by NO-GC to cGMP [132,133].

The heterodimer NO-GC1 consists of the subunits α_1_ and β_1_ and is expressed in most cell types and tissues [119,134]. The heterodimer NO-GC2, which consists of the subunits α_2_ and β_1_, is expressed in neuronal cells, in the placenta, in the spleen and in the uterus [119,122,123,135].

#### 2.3.2. The Maturation and Regulation of NO-GC Activity

In mammalian cells, 30 to 80% of the β-subunit of NO-GC is present in its heme-free form [136,137]. Once translated, the β-subunit of NO-GC is heme-free in cells and is termed Apo-NO-GCβ. It has been shown that cells exposed to physiological NO concentrations undergo a redistribution of heme into the subpopulations of the β-subunit of Apo-NO-GC [137,138]. The NO-controlled redistribution of cellular heme in the β-subunit of Apo-NO-GC depended on cell proteins glyceraldehyde-3-phosphate dehydrogenase (GAPDH) and heat shock protein 90 (Hsp90) [137,138]. Hsp90 is a cell chaperone that is responsible for the correct folding, stability, transport, and degradation of proteins [137,139].

In heme-free state, Apo-NO-GCβ forms a complex with Hsp90 [140,141]. GAPDH transports heme within cells and makes it available to the Apo-NO-GCβ-Hsp90 complex [142,143]. The insertion of heme into the β-subunit of NO-GC has been shown to result in the dissociation of Hsp90 from Apo-NO-GCβ-Hsp90 complex. This enables the α-subunit to bind to the β-subunit, allowing the mature αβ-heterodimer of NO-GC to be formed [140,141,144]. The mature αβ-heterodimer form of NO-GC can now respond to NO [137,141,144]. The binding of NO to the iron-containing (Fe^2+^) heme group of β_1_-subunit of NO-GC results in an increase in the catalytic activity of heterodimer NO-GC1 (α_1_β_1_-heterodimer) and NO-GC2 (α_2_β_1_-heterodimer), leading to the production of cyclic cGMP from GTP [17,18].

### 2.4. The NO-cGMP Signaling Cascade in Vascular Function

**Figure 3:**
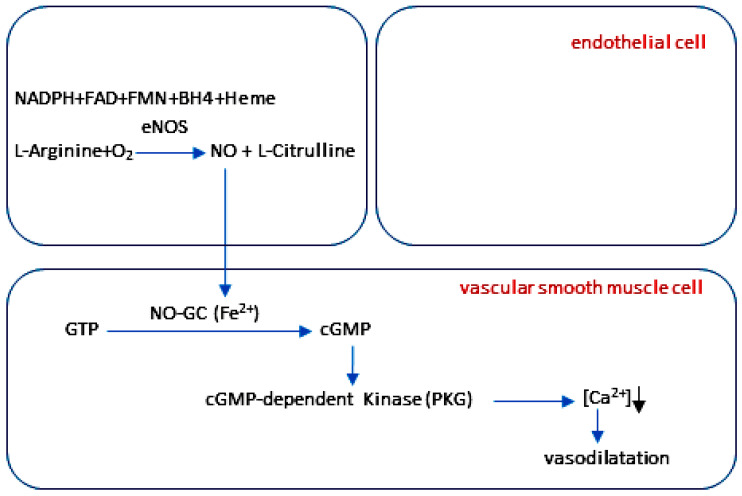
NO-cGMP signaling in blood vessels. In healthy endothelial cells, NO and L-citrulline are synthesised from L-arginine through the activity of eNOS. NO diffuses into the underlying smooth muscle cells and binds to NO-GC, inducing increased formation of cGMP. In the cell, this activates the downstream signaling cascade, which in turn activates cGMP-dependent protein kinases (PKG). The latter then mediate a decrease in intracellular Ca^2+^ concentrations (black arrow), leading to vasodilation.

In different cell types, the effects of NO vary depending on the interactions between the NO molecule and its derivatives and effectors, as well as the local redox environments [145]. In endothelial cells, NO is produced through the activity of eNOS [81,82]. Through the endothelial plasma membrane, NO diffuses across various cell types in the vascular wall (VSMCs) and blood (platelets) where it binds to NO-GC, resulting in the production of cGMP from GTP [17,18,114,146,147] (Figure 3). Through the downstream signaling cascade, cGMP interacts with cGMP-activated protein kinases (PKG1 and PKG2) [32,148], cGMP-regulated ion channels [149,150] and cGMP-regulated phosphodiesterases (PDEs) [52,151].

The NO-cGMP signaling cascade mediates vasodilation [18,19] (Figure 3), inhibits platelet aggregation [20,21,22,23] and leukocyte adhesion [25,27], and inhibits the proliferation and migration of VSMCs [28,29] exerting anti-inflammatory effects on cells in blood vessels [26,152]. Understanding the mechanism of action of NO-cGMP signaling cascades on a range of anti-inflammatory processes is therefore important for the treatment of inflammatory diseases in various organs, including the dental pulp.

## 3. NO and NO-cGMP Signaling in the Healthy Dental Pulp

### 3.1. The Healthy Dentin–Pulp Complex

In the dentin–pulp complex, the dental pulp contains odontoblasts [153,154], fibroblasts [155,156], nerve fibers with myelinated and unmyelinated peripheral glial cells [157,158,159], endothelium (endothelial cells) [12] with mural cells (VSMCs and pericytes) [120,159], immune cells such as macrophages [160,161], dendritic cells [6,162] and pulp glial [159,163] and mesenchymal [164,165] stem cells. As mesenchymal stem cells in the dental pulp originate from the neural crest during tooth development [166,167], they are also termed ectomesenchymal stem cells. In the dentin–pulp complex, the cell bodies of the odontoblasts are located in the peripheral pulp, while the processes of the odontoblasts extend within the dentinal tubules to the area near the enamel-dentin junction and the dentin-cement junction. In cell–cell and cell–matrix interactions, the cells of the dental pulp maintain homeostasis to ensure the physiological functions of the dentin–pulp complex. In the event of inflammation, the cells of the dental pulp are able to enable regeneration and repair of the dentin–pulp complex [4,5,7,153,168,169,170,171].

In an organ, the healthy endothelium fulfills the following functions: (i) induction of vasodilation, (ii) inhibition of coagulation (anti-coagulant) and induction of fibrinolysis (pro-fibrinolytic), (iii) inhibition of leukocyte adhesion and migration (anti-inflammatory), (iv) inhibition of platelet adhesion and aggregation (anti-thrombotic) and inhibition of the proliferation and migration of VSMCs (anti-hypertrophic) [32,152]. Therefore, clarifying the functions of the endothelial cells of the dental pulp under physiological and inflammatory conditions is of great importance for developing new treatment strategies for the therapy of caries.

### 3.2. The Regulation of Circulation by Intrapulpal Tissue Pressure in Healthy Dental Pulp

The dental pulp is surrounded by a rigid dentin matrix structure and is only open via an apical foramen, through which blood vessels and nerve fibres pass. This unique encasement of the dental pulp in a low-compliance environment is of great importance for pulp physiology and pulp blood flow due to the development of intrapulpal tissue pressure under physiological and inflammatory conditions [8,9,10,11,172]. In a physiologically intact dentin–pulp complex, the endothelium of the dental pulp is adapted to this unique structure. The endothelium of the dental pulp is able to respond adequately to tissue-specific signals in order to regulate homeostasis in the dentin–pulp complex under physiological conditions.

The cells of the vessel wall are sensitive to changes in their mechanical environment. Under physiological conditions, mechanical forces regulate signal transduction and gene expression in vascular cells in order to control the remodeling processes necessary for optimal function of the vascular wall. However, under inflammatory conditions, the cells undergo defective remodelling, impairing or losing homeostatic mechanisms [173]. Two biomechanical forces regulate mechanotransduction in blood vessels: These are cyclic stretching and shear stress. Cyclic stretching affects all cells in the vessel wall, while shear forces only affect the cells (endothelial cells) lining the vessel lumen. These mechanical forces trigger biological signals in the cells of the blood vessels [174]. The intrapulpal tissue pressure in normal pulp is higher in comparison to other organs [10,175]. This tissue-specific property of dental pulp has the ability to influence the mechanosensation of endothelial cells.

### 3.3. The Phosphorylation of eNOS at Ser1177 in the Endothelial Cells of Healthy Dental Pulp

It is known that endothelial cells of healthy dental pulp express eNOS [122,176,177]. The phosphorylation of eNOS has been identified as a critical regulatory mechanism for the activity of the enzyme in endothelial cells [47,108,178]. The phosphorylation of eNOS at Ser1177 results in an increase in the electron flux within the eNOS dimeric form, thus increasing eNOS activity and NO production. In contrast, the phosphorylation of eNOS at Thr495 has been demonstrated to decrease the electron flux within the eNOS dimer form, thereby inducing a decrease in eNOS activity [47,48]. In endothelial cells of the healthy human dental pulp, eNOS is phosphorylated strongly at Ser1177 but weakly at Thr495 [12]. In view of the results obtained, it is evident that eNOS is active in the endothelial cells of healthy dental pulp and produces NO through phosphorylation of the enzyme at Ser1177.

Endothelial cells are mechanosensitive [179,180], and it is known that shear stress increases the phosphorylation of eNOS at Ser1177 via the activation of Akt/PKB [48,107]. The activation of eNOS by phosphorylation of enzyme at Ser1177 is calcium-independent and increases the production of NO [48,107]. The phosphorylation of eNOS at Ser1177 in blood vessels of healthy dental pulp [12] can be explained by the unique structure of the dentin–pulp complex and the resulting intrapulpal tissue pressure. Since healthy dental pulp exhibits a higher physiological intrapulpal tissue pressure compared to other tissues [10,11,172], it is conceivable that this physiologically higher intrapulpal tissue pressure may induce an increase in shear stress in the endothelium by increasing friction between blood flow and the vessel wall. This, in turn, may lead to the phosphorylation of eNOS at Ser1177 in the endothelium of the healthy dental pulp, inducing the formation of NO. Following its formation, NO diffuses through the endothelial plasma membrane into the neighboring VSMCs. In VSMCs of the healthy dental pulp, the activation of NO-GC can lead to the dephosphorylation of the myosin light chain, resulting in vasodilation through the reduction in Ca^2+^ concentrations.

### 3.4. NO-GC and cGMP in Blood Vessels of Healthy Dental Pulp

In blood vessels of the human dental pulp, the expressions of the α_1_-, α_2_- and β_1_-subunits of NO-GC and localization of cGMP were detected [120,122,181]. In consideration of the results obtained, NO-GC1 (α_1_β_1_-heterodimer) appears to play a key regulatory role in the vasodilation process in dental pulp blood vessels by activating downstream NO-cGMP signaling cascades. The NO formed in endothelial cells of the healthy dental pulp diffuses into the underlying VSMCs activating NO-GC1 in these cells. The activation of the NO-GC1 enzyme in VSMCs induces the production of cGMP, which in turn leads to vasodilation in the blood vessels of healthy dental pulp.

### 3.5. The Role of the NO-cGMP Signaling in Maintaining Homeostasis of Healthy Dental Pulp

It has been well documented that, under physiological conditions, endothelial cells produce NO, which has been demonstrated to inhibit platelet aggregation, monocyte and leukocyte adhesion via a NO-cGMP signaling [27,50]. NO inhibits the proliferation and migration of VSMC via activation of the NO-cGMP signaling cascade [28,29]. The NO produced by endothelial cells diffuses to thrombocytes and leukocytes circulating in the blood, which express NO-GC. In platelets, NO activates the NO-GC leading to inhibition of platelet activation, adhesion and aggregation in blood vessels [25,27]. Activation of NO-GC in leukocytes induces a reduction in their adhesion to blood vessels [27].

In addition to vasodilation, the anti-inflammatory effect of the NO-cGMP signaling cascade could also play an important role in the homeostasis of the dentin–pulp complex. Under physiological conditions, NO may regulate its anti-inflammatory effect by activating NO-GC via the NO-cGMP signaling cascade in cells of the dental pulp. The inhibitory effect of the NO-cGMP signaling cascade on platelet activation, adhesion, and aggregation, as well as its ability to inhibit leukocyte adhesion in blood vessels, are the underlying mechanisms that could also occur in the blood vessels of the dental pulp to maintain homeostasis of the dentin–pulp complex under physiological conditions.

## 4. NO and NO-cGMP Signaling in Endothelial Dysfunction

The balance between NO production through eNOS activity and the uncoupling of eNOS due to higher concentrations of ROS and RNS determines endothelial function [32,50,89,182]. Under normal conditions, the production of ROS and RNS occurs at physiological concentrations, which is necessary for normal cell function [183,184]. Inflammation has been shown to induce an increase in the production of ROS and RNS, which can result in eNOS uncoupling and, consequently, endothelial dysfunction [89,90,185,186]. ROS and RNS include superoxide radicals (O_2_^−^), hydrogen peroxide (H_2_O_2_), hydroxyl radicals (HO·), peroxyl radicals (HO_2_), nitrogen monoxide radicals (·NO), nitrogen dioxide radicals (NO_2_), and peroxynitrite (ONOO^−^) [183,184]. Endothelial dysfunction is associated with uncoupling eNOS, reduced bioavailability of NO and oxidized NO-GC, resulting in decreased production of cGMP due to higher concentrations of ROS and RNS [32,50].

### 4.1. The Uncoupled eNOS

In inflammation ROS and RNS have been shown to lead to an uncoupling of eNOS through various mechanisms [89,90]. These include the oxidation of BH4 to BH2 [187], the oxidative disruption of the zinc–sulfur complex of eNOS dimer [188], the phosphorylation of the enzyme at Thr495 [189,190], S-glutathionylation of the enzyme [191,192], reduced substrate availability of the enzyme [13,193], and the effects of the endogenous inhibitor of eNOS, asymmetric dimethylarginine (ADMA) [194,195].

#### 4.1.1. Uncoupled eNOS by Oxidative Depletion of Tetrahydrobiopterin (BH4)

**Figure 4:**
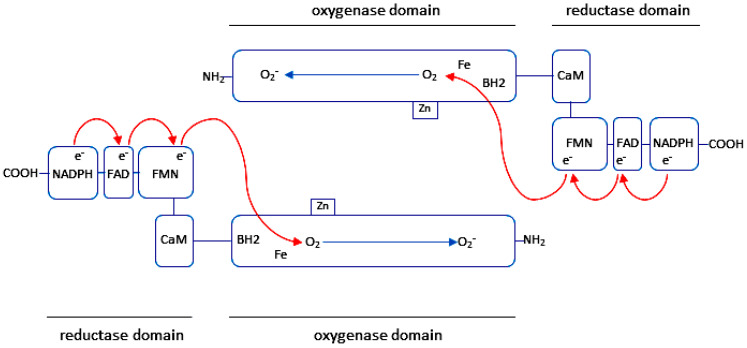
Uncoupled eNOS and formation of O_2_^−^ by eNOS in monomer form. NO is produced by eNOS in dimer form. However, eNOS is not able to form NO in monomer form. In the presence of inflammation, the dimer form of eNOS can undergo uncoupling into its two monomer forms, a process triggered by the presence of ROS and RNS. The mechanisms that cause eNOS uncoupling include the oxidation of the NOS cofactor BH4 to BH2, reduced L-arginine levels, the accumulation of the endogenous NOS inhibitor ADMA, and S-glutathionylation of eNOS. In the uncoupled form of eNOS, electron transfer occurs from NADPH via FAD and FMN of the reductase domain to molecular oxygen (O_2_) in the oxygenase domain of the same monomer. This results in the formation of superoxide anions (O_2_^−^). The illustration was modified from [32].

Tetrahydrobiopterin (BH4) is synthesised from GTP by the enzyme guanosine 5′-triphosphate cyclohydrolase I (GTPCH) [196,197,198,199]. BH4 is an essential cofactor for eNOS to regulate electron flow within the eNOS dimer and thus enable the formation of NO [47,50,89,200]. The uncoupling of eNOS is associated with a BH4 deficiency in an environment with elevated concentrations of O_2_^−^ and ONOO^−^ [187,199,201,202,203]. O_2_^−^ and ONOO^−^ induce the activation of the ubiquitin-proteasome cascade, which consequently results in the degradation of GTPCH. The degradation of GTPCH has been demonstrated to result in a rapid depletion of BH4 [199,202]. In this reaction, ONOO^−^ oxidizes BH4 to an intermediate product, BH3, which is further oxidized to BH2 [187], resulting in uncoupling of eNOS [32,50,89,187,199,200,201] (Figure 4).

#### 4.1.2. Uncoupled eNOS by Oxidative Disruption of the Zinc-Sulfur Complex

The oxygenase domain of eNOS contains binding sites for BH4, heme iron, and L-arginine. The zinc thiolate (ZnS4) cluster in the eNOS oxygenase domain, formed from one zinc ion and two cysteine residues from each monomer, is responsible for maintaining the integrity of the BH4 binding site in eNOS [50,204]. The stabilization of the eNOS dimer by the ZnS4 center is an essential prerequisite for the catalytic activity of eNOS [204]. A mutation in the ZnS4 cluster has been demonstrated to prevent the binding of zinc, BH4, or L-arginine and to abolish the activity of eNOS [50,205]. It has been demonstrated that oxidation of the zinc thiolate cluster by ONOO^−^ leads to dimer dissociation of eNOS (uncoupling of eNOS), resulting in the formation of O_2_^−^ instead of NO [32,89,188,206] (Figure 4).

#### 4.1.3. Uncoupled eNOS by Phosphorylation of Enzyme at Thr495

The activity of eNOS is increased by phosphorylation of the enzyme at Ser1177, which is mediated by Akt/PKB activity. It has been demonstrated that the phosphorylation of eNOS at Ser1177 is Ca^2+^-independent, resulting in the formation of NO [107]. In contrast, it has been shown that eNOS activity decreases through phosphorylation of the enzyme at Thr495, which is mediated by protein kinase C (PKC) activity [189,190]. ROS and RNS have been verified to induce phosphorylation of eNOS at Thr495, leading to eNOS uncoupling [189,190]. This uncoupling is a consequence of the action of ONOO^−^, wherein uncoupled eNOS forms O_2_^−^ instead of NO [189,190].

#### 4.1.4. Uncoupled eNOS by S-Glutathionylation of the Enzyme at Cys689 and Cys908

Under oxidative (ROS) and nitrosative stress (RNS), the activity of eNOS is negatively regulated by S-glutathionylation at the cysteine residues Cys689 and Cys908 in the reductase domain of the enzyme, leading to the uncoupling of eNOS and the formation of O_2_^−^ in endothelial cells [191,192]. The cysteine residues Cys689 and Cys908 located at the interface between the FMN and FAD binding domains undergo S-glutathionylation, resulting in an uncoupling mechanism of eNOS that leads to the release of electrons to molecular oxygen with the formation of O_2_^−^ at the reductase domain [192,207]. The S-glutathionylation of eNOS catalyzed by glutaredoxin-1 is a reversible process [207].

#### 4.1.5. Uncoupled eNOS by Reduced Substrate Availability

L-arginine is the endogenous substrate for all forms of NOS, including eNOS. A reduced availability of L-arginine for eNOS can lead to uncoupling of eNOS [13,193]. L-arginine is also a substrate for the enzymes arginase I and II, which catalyze the hydrolysis of L-arginine to urea and ornithine [208,209]. Therefore, an L-arginine deficiency may result from increased arginase activity [210]. Increased ROS and RNS production under pathological conditions has been identified as the primary cause of L-arginine deficiency in blood vessels [13,211]. When endothelial arginase activity increases due to ROS and RNS, arginase competes with eNOS for the common substrate, which can lead to eNOS uncoupling [212,213,214,215]. A further reason for the decrease in L-arginine levels is the induction of iNOS [214]. Under physiological conditions, the enzyme iNOS can only be expressed to a minimal extent. However, under inflammatory conditions, iNOS is continuously expressed and produces large amounts of NO. Therefore, iNOS competes with eNOS for the common substrate L-arginine, which can result in uncoupling of eNOS [214,216].

#### 4.1.6. Uncoupled eNOS by Asymmetric Dimethyl Arginine (ADMA)

Endogenous inhibitor of eNOS, asymmetrical dimethyl arginine (ADMA), has been shown to trigger the uncoupling of eNOS [50,194,195,217]. In response to the effects of ROS and RNS, ADMA is produced in higher levels [89,218]. ROS and RNS activate the enzyme protein arginine methyl transferase type I, which catalyzes the formation of ADMA. ROS and RNS inhibit the enzyme dimethylarginine dimethylamine hydrolase, which hydrolyzes ADMA, leading to increased ADMA levels even under oxidative stress [89,218]. This, in turn, induces uncoupling of eNOS [50,89,218,219].

### 4.2. Desensitization of NO-GC to NO

The desensitization of NO-GC to NO is triggered either by the loss of the heme group or by thiol oxidation and S-nitrosylation as a result of the effects of ROS and RNS [90,133].

#### 4.2.1. Heme Iron Oxidation in NO-GC

The redox state of the heme group in NO-GC is a decisive factor in its activation by binding to NO. In its native form, NO-GC requires a bound Fe^2+^-heme group in order to be sensitive to NO [111,220]. ROS and RNS produced during inflammation, leading to the conversion of Fe^2+^ to Fe^3+^ in the heme group of NO-GC [220,221,222]. In many cases, this results in the loss of the heme group from NO-GC, forming heme-free apo-NO-GC. Both the oxidized form of NO-GC with Fe^3+^-heme and apo-NO-GC are insensitive to NO, resulting in NO-GC exhibiting no activity even in the presence of endogenous and exogenous NO [111,131,220,223].

#### 4.2.2. Thiol Oxidation and S-Nitrosylation of NO-GC

The binding of an NO group to the thiol side chain of cysteine residues in proteins is referred to as S-nitrosylation. S-nitrosylation is defined as an NO-dependent post-translational modification of free thiol cysteines with the capacity to alter the function of proteins [224,225]. NO-GC is inactivated by thiol oxidation and S-nitrosylation [130,218,226,227]. The attachment of an NO group to the thiol side chain of cysteine residues in NO-GC leads to desensitization of NO-GC to NO [218,226]. It has been found that the desensitization of NO-GC is concentration- and time-dependent on exposure to S-nitrosocysteine, which can be explained by the NO tolerance of NO-GC [228,229].

## 5. NO and NO-cGMP Signaling in Endothelial Dysfunction of Inflamed Dental Pulp

### 5.1. Inflammation of the Dental Pulp

In the event of carious lesions extending beyond the enamel-dentin or cementum-dentin junctions, bacteria have the ability to penetrate the exposed dentinal tubules and enter the dental pulp, inducing inflammation [181,230]. The pathogens contain repetitive patterns in their cell membranes that enable immune cells or other cells to recognize them as foreign. These repetitive patterns of bacterial components are known as pathogen-associated molecular patterns (PAMPs). In the earlier phase of the carious lesion, the odontoblasts, the fibroblasts, endothelial cells, stem cells, and immune cells of the dental pulp recognize PAMPs via pattern-recognition receptors (PRRs) [5,168]. The PRRs, Toll-like receptors (TLRs), and nucleotide-binding oligomerization domain proteins (NOD) 1 and 2 are expressed on odontoblasts, fibroblasts, pulp stem cells, and in endothelial cells [5,168]. The highly selective endothelial barrier is essential for maintaining fluid homeostasis in tissue to support normal organ function [231,232]. A hallmark of the endothelium during inflammation is an increased permeability, resulting in the loss of barrier function and subsequent tissue oedema [231,232].

The bacteria induce inflammation in the dental pulp through severe vasodilation and vascular permeability, which can lead to increased plasma extravasation [8,233,234,235]. In the inflamed dental pulp, the initial response to bacterial infection is the secretion of toxic substances by neutrophil granulocytes to eliminate bacteria. The macrophages then initiate the process of bacterial phagocytosis. The transcription factor nuclear factor kappa B (NF-κB) is a crucial component of the immune response, being found to be upregulated in immune cells during inflammation [232,236]. The activation of NF-κB is regulated by the cytokines interleukin-6 (IL-6), tumour necrosis factor-alpha (TNF-α), and IL-8 [232,236]. The nuclear translocation of NF-κB leads to increased production of cytokines and chemokines (IL-1α and IL-1β; TNF-α; IL-4, IL-6, IL-8, IL-10), which trigger cellular immune responses in the inflamed dental pulp [5,168]. In response to inflammatory cytokines, endothelial cells are activated and express adhesion molecules (selectins, integrins, intercellular adhesion molecules (ICAMs) and vascular cell adhesion molecule-1 (VCAM-1)) [232,236]. This, in turn, triggers the process of recruitment of immune cells to the site of inflammation in the dental pulp. The number of neutrophils, macrophages, dendritic cells, mast cells, T lymphocytes and B lymphocytes increases in the area of inflammation near a carious lesion [160,181,234,237,238]. In comparison with healthy human dental pulp, inflamed human dental pulp has been shown to exhibit higher concentrations of ROS [12] and RNS [234]. At higher concentrations, ROS and RNS cause cellular damage due to their deleterious effects on DNA, proteins, and lipids in cells of the dentin–pulp complex [5,12,168,230,234].

The dental pulp is located in a rigid, mineralized extracellular dentin matrix, which limits its ability to expand in response to inflammation caused by caries. This unique structure exerts a significant influence on the functionality of endothelial cells under inflammatory conditions, given the crucial role of pulp circulation in the regeneration and repair of the dentin–pulp complex.

### 5.2. The Regulation of Circulation by Intrapulpal Tissue Pressure in Inflamed Dental Pulp

The dental pulp is located within a chamber of mineralized dentin matrix that only opens to blood vessels and nerve fibres via the apical foramen. This unique structural morphology limits the ability of the dental pulp to expand and adapt in response to inflammation caused by mechanical, chemical, thermal, and bacterial stimuli [8,10,11,175]. Following a carious lesion, local inflammation develops in the dental pulp. The vasodilation and increased capillary permeability caused by the inflammation lead to increased plasma extravasation from the blood vessels into the tissue, which in turn results in a steady increase in tissue pressure in the dental pulp. As intrapulpal tissue pressure increases, the blood vessels can gradually become strangulated, which can lead to a reduction in blood flow and thus to ischemia of the dental pulp [8,10,11,175].

Despite the absence of collateral circulation in the dental pulp, its micromorphological and physiological properties have the capacity to compensate for the elevated tissue pressure that occurs during inflammation [10,11]. For instance, the dental pulp contains shunt vessels that form a direct link between the arterioles and venules [2,239]. When intrapulpal pressure rises, the shunt vessels open to reduce it, thereby maintaining blood flow in the dental pulp [2,172]. Increased lymph flow in the dental pulp may also counteract increased hydrostatic dental pulp tissue pressure [11,175]. It has been shown that the dense extracellular matrix (ECM) in the dental pulp limits intrapulpal pressure at the site of irritation [175]. Initially, the increased pressure causes the thin-walled venules in the microenvironment of the affected pulp tissue to collapse due to increased vasodilation, increased permeability, and plasma extravasation. Only when the structural integrity of the ECM in the dental pulp is lost due to massive inflammation can the increased tissue pressure spread and lead to compression of the blood vessels at the apical foramen. This results in reduced blood flow to the pulp [10,240].

### 5.3. The Formation of ROS and RNS in Inflamed Dental Pulp

At physiological concentrations, ROS and RNS are essential for regulating normal cell functions [183,184] such as maintaining vascular tone through the endothelium, promoting angiogenesis, and acute inflammatory responses to combat invading pathogens [232,241]. Nevertheless, the continuous elevation of ROS and RNS under inflammatory conditions leads to reactions with cell lipids, proteins, and nucleic acids, resulting in cell damage [90,186,242,243].

The biological sources of the superoxide anion (O_2_^−^), a precursor of ROS and RNS, are xanthine oxidase (XO), NADPH oxidases (NOX), uncoupled nitric oxide synthases (eNOS, nNOS and iNOS) and the mitochondrial respiratory chain [186,211,219,244]. In view of these results obtained under inflammatory conditions in blood vessels, XO, NADPH oxidases, uncoupled eNOS, nNOS, iNOS and the mitochondrial respiratory chain may be considered as sources of O_2_^−^ formation in blood vessels of the inflamed dental pulp. In comparison to the healthy human dental pulp, a significantly increased phosphorylation of eNOS at Thr495 in blood vessels of inflamed human dental pulp indicates uncoupling of eNOS and the formation of O_2_^−^ instead of NO in blood vessels of inflamed human dental pulp [12].

The activity of iNOS is primarily regulated at the transcriptional level [47,92]. The transcription factor NF-κB plays a crucial role in inducing endogenous *iNOS* gene expression in response to bacterial lipopolysaccharide (LPS) [92,93]. In the event of a carious lesion, LPS activates the NF-κB pathway in dental pulp cells leading to the production of cytokines and chemokines, which cause inflammation in the dental pulp [5,6,168,170]. In inflammation, iNOS becomes permanently active in endothelial cells and VSMCs, thereby continuously generating NO [100,245]. Higher expression of iNOS has been detected in inflamed dental pulp [177,234,246,247]. The expression of iNOS in inflamed dental pulp is regulated by transcriptional activation in response to inflammation and cytokine production. The sustained activity of iNOS in inflamed dental pulp results in elevated levels of NO formation. In the presence of inflammation, NO reacts with O_2_^−^ to form the highly toxic compound ONOO^−^ [100,248]. The biological existence of ONOO^−^ is demonstrated specifically by detecting 3-nitrotyrosine (3NT) [249,250] in healthy and inflamed tissues using immunohistochemistry and immunoblotting [251,252,253,254]. In healthy dental pulp, 3NT was detected in low levels; in contrast, inflammation of the dental pulp caused by caries led to higher expression of 3NT [234]. The elevated levels of 3NT are the result of increased formation of ONOO^−^ in inflamed human dental pulp.

### 5.4. Uncoupled eNOS and Endothelial Dysfunction in Inflamed Dental Pulp

The inflamed endothelial cells have the ability to produce ROS and RNS in higher levels, which in turn are mediated by the mitochondrial respiratory chain, NOX (in particular NOX2 and NOX4), XO and uncoupled eNOS [232,255]. In inflammation, ROS and RNS are formed in different subcellular compartments of a cell through the activity of various enzymes. ROS and RNS are generated in the cytoplasm, mitochondria, peroxisomes and endoplasmic reticulum during inflammation [218,256]. In the cytosol, ROS are formed by NADPH oxidases [256]. The enzymes nNOS, eNOS and iNOS are sources of ROS formation in the cytosol during inflammation. Uncoupling nNOS, eNOS and iNOS during inflammation leads to a dysregulated NO response, in which NO reacts with O_2_^−^ to form ONOO^−^ [89,248]. Uncoupled eNOS is a hallmark of inflammation in blood vessels [89,257]. Inflammation of the human dental pulp, in which O_2_^−^ [12] and ONOO^−^ [234] are produced in excessive amounts, leads to uncoupling of eNOS. This results in endothelial dysfunction of the inflamed dental pulp. Endothelial dysfunction in inflamed dental pulp is associated with reduced formation as well as reduced bioavailability of NO.

In nerve fibers and in odontoblasts of the dental pulp, nNOS is expressed [122]. As nNOS is expressed during inflammation, a process in which the enzyme can become uncoupled [188,258], it is possible that uncoupled nNOS could also be the source of O_2_^−^ in inflamed dental pulp. In inflamed dental pulp, iNOS is known to be activated simultaneously in immune cells and in the endothelium, resulting in the release of substantial quantities of NO [177,234]. The reaction between NO and O_2_^−^ results in the formation of ONOO^−^, which has been detected in increased concentrations within inflamed dental pulp by 3NT [234]. The formation of O_2_^−^ [12] and ONOO^−^ [234] in inflamed human dental pulp may play a critical role in the development of endothelial dysfunction by uncoupling eNOS in endothelial cells.

#### 5.4.1. Uncoupled eNOS by Phosphorylation of eNOS at Thr495 in the Endothelial Cells of Inflamed Dental Pulp

The activity of eNOS in endothelial cells increased by phosphorylation of the enzyme at Ser1177 and decreased at Thr495 [47,48,259]. In the endothelium of the inflamed dental pulp, eNOS was found to be phosphorylated weakly at Ser1177 but strongly at Thr495 [12]. These results indicate that eNOS activity is reduced by phosphorylation of the enzyme at Thr495 in inflamed dental pulp.

The unique structure of the dental pulp could play a crucial role in the phosphorylation of eNOS at Thr495 under inflammatory conditions. Inflammation of the dental pulp results in elevated intrapulpal tissue pressure due to increased vasodilatation, permeability and plasma extravasation in blood vessels of the dental pulp. This, in turn, has been shown to result in a significant reduction in blood flow through the small apical foramina to the dental pulp, which is surrounded by a rigid dentin matrix [8,10,11]. The reduced blood flow in this low compliance [11,260] indicates a reduction in shear forces on the blood vessels in the inflamed dental pulp. The decrease in eNOS activity due to reduced phosphorylation of the enzyme at Ser1177 and increased phosphorylation of the enzyme at Thr495 is attributable to a lack of shear stress in the pulp blood vessels caused by reduced blood flow in the inflamed dental pulp. This assumption is supported by findings showing that low shear stress induced phosphorylation of eNOS at Thr495 through activation of MAPK ERK1/2 [261,262]. It has also been found that low shear stress with inflammation in endothelial cells is associated with the formation of ROS, which reduces the activity of eNOS through phosphorylation of the enzyme at Thr495 [261,262,263,264].

The phosphorylation of eNOS at Thr495, which is mediated by PKC, contributes to the uncoupling eNOS and production of O_2_^−^ instead NO [189,190]. A significant increase in the phosphorylation of eNOS at Thr495 was detected in blood vessels of inflamed human dental pulp in comparison to healthy dental pulp [12]. The results obtained indicate the uncoupling of eNOS and the formation of O_2_^−^ instead of NO by phosphorylation of the enzyme at Thr495 in inflamed blood vessels of the human dental pulp following a carious lesion, which in all cases triggers inflammation in the dental pulp when the dentinal tubules are exposed by the lesion.

#### 5.4.2. The Uncoupling of eNOS Through the Oxidation of BH4 and ZnCys4 in Inflamed Dental Pulp

The cofactor BH4 and the ZnCys4 complex, which connect the two monomers and enable dimerisation of eNOS, are crucial for dimerisation of eNOS and for formation of NO [188,196,198,265]. Through redox regulation, the ZnCys4 binding region of the eNOS dimer is oxidized by ROS and RNS, particularly by ONOO^−^. This leads to a loss of eNOS dimerisation and subsequent uncoupling of eNOS [89,188]. Inflammation of the blood vessels has been demonstrated to induce the oxidation of BH4 to BH2, which in turn can lead to the uncoupling of eNOS [196,197,198,199,201,266]. The uncoupling of eNOS through the oxidation of BH4 to BH2 and through the oxidation of ZnCys4 by ROS and RNS leads to the formation of O_2_^−^ instead of NO in endothelial cells [32,196,198,200,201]. In consideration of these results, it can be assumed that the formation of ONOO^−^ [234] in inflamed human dental pulp induces uncoupling of eNOS through the oxidation of BH4 to BH2 and the oxidation of ZnCys4 in the blood vessels of the pulp, which may lead to the production of O_2_^−^ in the inflamed dental pulp. It is also expected that eNOS in endothelial cells of the inflamed dental pulp can be uncoupled by S-glutathionylation, whereby the enzyme reversibly switches from NO to O_2_^−^ production.

#### 5.4.3. The Uncoupling of eNOS Due to Competition with iNOS for the Common Substrate L-Arginine in Inflamed Dental Pulp

The sustained expression of iNOS is regulated by transcriptional activation in response to inflammation and cytokine production. Therefore, iNOS is highly dependent on the availability of the substrate L-arginine, which is also used as a substrate by eNOS. Therefore, iNOS competes with eNOS for the common substrate L-arginine, which can result in uncoupling of eNOS [214,216]. In inflamed dental pulp, iNOS shows higher activity [177,234,246,247]. It can therefore be assumed that active iNOS in inflamed dental pulp competes with eNOS for the substrate L-arginine, which could lead to an insufficient amount of L-arginine, triggering an uncoupling of eNOS.

#### 5.4.4. The Uncoupling of eNOS Due to Increased Formation of the Endogenous eNOS Inhibitor ADMA in Inflamed Dental Pulp

In response to the effects of ROS and RNS, ADMA (an endogenous inhibitor of eNOS) is produced in higher levels [89,218]. It has been reported that ADMA triggers the uncoupling of eNOS [50,194,195,217]. Higher concentrations of ROS and RNS have been detected in inflamed dental pulp [12,50]. Therefore, it is to be expected that the concentrations of the endogenous eNOS inhibitor ADMA may be elevated in inflamed dental pulp. In inflamed dental pulp, ADMA can uncouple the enzyme eNOS, resulting in the formation of O_2_ rather than NO by uncoupled eNOS.

### 5.5. Oxidized NO-GC and Endothelial Dysfunction in Inflamed Dental Pulp

The inflammation-dependent expression of mediators and factors in activated endothelial cells, VSMCs, macrophages, and lymphocytes in the inflamed dental pulp may lead to the formation of higher concentrations of cytokines, growth factors, ROS, and RNS within the blood vessel wall of the inflamed dental pulp [230]. The formation of O_2_^−^ [12] and ONOO^−^ [234] in inflamed human dental pulp may play a critical role in the development of endothelial dysfunction by oxidizing NO-GC in VSMCs.

It has been reported that NO-GC transcription and NO-GC mRNA stability can be affected by ROS and RNS under pathological conditions [267]. Indeed, at the protein level, a decrease in the α_1_-, β_1_-, and α_2_-subunits of NO-GC has been detected in cells of inflamed human dental pulp, while inflammation induced a significantly increased expression of ONOO^−^ in human dental pulp cells [234]. It has been established that ONOO^−^ significantly reduces the catalytic enzyme activity of NO-GC [268]. The downregulation of expression and decrease in activity of NO-GC by ROS and RNS under pathological conditions may be explained by transcription inhibition, mRNA destabilization, and protein destabilization [267].

In the event of inflammation, elevated ROS and RNS levels trigger the oxidation of Fe^2+^ to Fe^3+^ within the heme group of NO-GC, making NO-GC insensitive to NO [220,221,269]. In inflamed dental pulp, endothelial dysfunction is characterized by reduced formation of NO in endothelial cells due to uncoupled eNOS [12]. As a consequence of elevated concentrations of ONOO^−^ in inflamed human dental pulp [234], it can be assumed that NO-GC becomes NO-insensitive, due to the oxidation of Fe^2+^ to Fe^3+^ by ONOO^−^ in heme group of NO-GC in the inflamed dental pulp. Given the insensitivity of NO-GC to NO in inflamed dental pulp, the anti-inflammatory role of NO in blood vessels of the inflamed dental pulp is not able to be regulated via the NO-GC.

NO has been demonstrated to inhibit the proliferation and migration of VSMC by activating the NO/NO-GC/cGMP signaling cascade [28,29]. It has been shown that the loss of NO-GC in platelets can result in enhanced adhesion of leukocytes to endothelial cells, the development of atherosclerotic plaques, and inflammation in blood vessels [23]. In addition, it has been reported that reduced NO/NO-GC/cGMP signaling in blood vessels is associated with neointimal proliferation, leading to endothelial dysfunction during inflammation of the blood vessels [270]. Increased platelet aggregation and enhanced adhesion of monocytes and leukocytes are to be expected in the blood vessels of inflamed dental pulp due to oxidized NO-GC. This can exacerbate inflammation in the dental pulp, leading to a transition from the acute inflammatory phase to a chronic phase. In the case of chronic inflammation, the dental pulp has a reduced potential for regeneration and repair.

### 5.6. The Critical Role of Endothelial Dysfunction in Inflammation of the Dental Pulp

Healthy endothelial cells produce vasodilators and anticoagulants (NO, prostacyclin), which have been shown to reduce platelet aggregation as well as the adhesion of monocytes and leukocytes [27,50]. NO inhibits the proliferation and migration of VSMCs in healthy tissues via activation of the NO-GC [28,29,31]. The NO-cGMP signaling cascade in the cells of the dental pulp [12,122,234,271] indicates that the NO-cGMP signaling cascade, with its anti-inflammatory effects, maintains the balance of the dentin–pulp complex. However, in the event of inflammation, endothelial cells release factors and inflammatory mediators (thromboxane and endothelin-1) due to endothelial dysfunction, which enhance platelet aggregation, monocyte adhesion, vasoconstriction and VSMC proliferation [27,272].

In the case of inflammation, the inflammatory mediators IL-1, TNF, endotoxin, and oxidized lipoproteins (ox-LDL) and biomechanical stimuli caused by impaired blood flow, lead to endothelial activation [15,27]. The inflammatory mediators primarily activate NF-κB, which leads to the expression of additional proinflammatory mediators within the endothelial cell [15,27]. In endothelial cells, adhesion molecules (vascular cell adhesion molecule-1 [VCAM-1]), chemokines (monocyte chemotactic protein [MCP]-1) and prothrombotic mediators (tissue factor [TF]), von Willebrand factor [vWF] and plasminogen activator inhibitor [PAI]-1) are then expressed [15,27]. The inflammation-dependent expression of adhesion molecules, chemokines, chemotactic proteins, and prothrombotic mediators promotes the selective recruitment of monocytes and different subpopulations of T lymphocytes, which accumulate in the subendothelial space [15,27]. In the inflamed dental pulp, endothelial cells increase their expression of adhesion molecules, including E-selectin, ICAM-1 and VCAM-1 [6,273] indicating an endothelial dysfunction in the inflamed dental pulp. Endothelial dysfunction in the dental pulp leads to stronger adhesion of circulating leukocytes and/or activated thrombocytes, as well as increased migration of immune cells into the dental pulp. In dental pulp inflammation, endothelial cells release less NO due to reduced eNOS activity [12]. In chronic dental pulp inflammation accompanied by endothelial dysfunction, endothelial cells secrete more VEGF, which, under inflammatory conditions in the dental pulp, increases endothelial cell proliferation to regulate angiogenesis.

The formation of O_2_^−^ [12] and ONOO^−^ [234] in inflamed human dental pulp may play a critical role in the development of endothelial dysfunction by uncoupling eNOS in endothelial cells and oxidizing NO-GC in VSMCs. The presence of high concentrations of ONOO^−^ in the dental pulp can induce a series of chemical reactions, including lipid peroxidation, protein oxidation, and nitration. In cells of the dental pulp, ONOO^−^ may cause irreversible damage to the respiratory chain, inhibition of ATP synthesis, cytochrome c release, and induction of caspase-dependent apoptosis. These reactions subsequently may result in the inactivation of enzymes and, ultimately, cell necrosis.

In addition to the endothelial dysfunction of the dental pulp caused by inflammation, the unique structure of the dental pulp is also of crucial importance in the course of inflammation in the dental pulp. The dental pulp is located within a rigid, mineralized extracellular dentin matrix structure that can only expand to a limited extent in the event of inflammation caused by caries. In many cases, this low compliance can result in reduced blood flow to the dental pulp during inflammation, which is associated with increased intrapulpal tissue pressure [8,9,10,11,172]. This, in turn, can promote a transition from acute to chronic inflammation. Inflammation of the dental pulp may be sustained in a chronic inflammatory state, which significantly impairs the regeneration and repair of the dental pulp. It is therefore critical to consider the treatment of endothelial dysfunction in the dental pulp when treating caries.

## 6. Targeting the NO-cGMP Signaling in the Treatment of Endothelial Dysfunction in Inflamed Dental Pulp

### 6.1. The Significance of Endothelial Dysfunction for the Treatment of Caries

The formation of dentin matrix is dependent on the vitality of the dental pulp [274] and the integrity of its endothelial function [12]. The objective of caries treatment is to induce the formation of reactive and reparative tertiary dentin matrix in response to carious lesions [4,5,274,275]. Reactive tertiary dentin matrix is formed by terminally differentiated odontoblasts [4,154,170,274]. In contrast, reparative tertiary dentin matrix is formed by odontoblast-like cells that differentiate from ectomesenchymal dental pulp stem cells in response to the degradation of terminally differentiated odontoblasts during dental pulp inflammation [4,154,163,170].

#### 6.1.1. Dentin Matrix Formation Under Healthy and Inflammatory Conditions in the Dentin–Pulp Complex

Odontoblasts are cells that form the dentin matrix during tooth development. Under physiological conditions, odontoblasts form the primary and secondary dentin matrix. Terminally differentiated odontoblasts form the primary dentin matrix during dental organ development. After the dental organ erupts, enters occlusion, and the apical foramen reaches its physiological diameter, terminally differentiated odontoblasts slowly form the secondary dentin matrix throughout life in response to occlusal forces [154,276]. Following carious lesions, odontoblasts form the tertiary dentin matrix to protect the dental pulp against bacteria and their metabolic products [276,277]. In response to carious lesions in dentin, terminally differentiated odontoblasts form a reactive tertiary dentin matrix [278,279,280]. When odontoblasts are degraded at their site, and dental pulp is exposed by a deep carious lesion, pulp stem cells differentiate into odontoblast-like cells that form a reparative tertiary dentin matrix [279,281]. Following deep carious lesions, odontoblast-like cells differentiate from ectomesenchymal and glial stem cells, as well as from pericytes in the dental pulp [159,163,167,282].

#### 6.1.2. The Role of Endothelial Dysfunction in the Regenerative Functions of Odontoblasts

The dental pulp is highly vascularized [2,283,284]. The blood vessels of the dental pulp pass through the apical foramen into the root pulp, accompanied by nerve fibres. The pulp blood vessels run centrally through the dental pulp and branch into arterioles. These arterioles then form a capillary network below the odontoblast layer at the periphery of the pulp through further fine branches [285,286]. The endothelium of the capillaries in the dental pulp is continuous beneath the odontoblast layer [3,287], which suggests higher metabolic activity and increased ion exchange between endothelial cells and odontoblasts [3,239]. Studies using ultrastructural electron microscopy have shown that ischaemia can cause changes in the nuclei and organelles of odontoblast cells in human dental pulp [288]. Following one hour of ischaemia in the dental pulp, chromatin clumping and irregular changes in the nuclear membrane structure were observed in odontoblast cell nuclei. The cytoplasm of the odontoblasts also contained numerous swollen mitochondria. By contrast, no cellular changes were observed in the odontoblasts of the control groups [288]. These results show that the functions of odontoblasts are regulated depending on the circulation of the dental pulp.

It has been reported that the formation of tertiary dentin matrix in response to carious lesions depends on the intact endothelial function of human dental pulp [12]. Endothelial dysfunction resulting from the uncoupling of eNOS, the phosphorylation of eNOS at Thr495, and the formation of ROS and RNS in inflamed human dental pulp can significantly reduce the regenerative capacity of odontoblasts. The treatment of endothelial dysfunction in inflamed dental pulp can maintain pulp vitality in balance and improve the function of dental pulp cells, thereby enabling the formation of a reactive or reparative tertiary dentin matrix against carious lesions. The use of NO-GC stimulators and activators in combination with PDE5i in dentin wounds or in pulp capping materials during caries treatment can increase the intracellullar cGMP level in the cells of the blood vessels of inflamed dental pulp, promoting the regeneration or repair potential of odontoblasts and odontoblast-like cells in inflamed dental pulp.

### 6.2. NO as a Therapeutic Target in Endothelial Dysfunction of Inflamed Dental Pulp

The mechanisms that cause eNOS uncoupling include the oxidation of the NOS cofactor BH4 to BH2, reduced L-arginine levels, the accumulation of the endogenous NOS inhibitor ADMA, and S-glutathionylation of eNOS [89,185,206,218]. NO donors (e.g., organic nitrates) that target the NO signaling pathway are used to treat cardiovascular diseases. However, certain limitations have been identified in the use of organic nitrates [33,37,206,229]. The following mechanisms could be responsible for this [33,289]: One mechanism that has been identified is the oxidation of the heme group of NO-GC under inflammatory conditions, which has been shown to render NO-GC insensitive to NO [220,223]. Another mechanism is tolerance to organic nitrates, which develops after long-term treatment with organic nitrates [34,289,290]. The third mechanism is the formation of ONOO^−^ in inflammation. In inflammation, O_2_ is produced by a number of enzymes. Although organic nitrates increase NO levels, under inflammatory conditions, this NO reacts with O_2_^−^ to form ONOO^−^ [256,291]. ONOO^−^ interacts with proteins and lipids, resulting in the disruption of mitochondrial function and the induction of DNA damage [292,293].

#### 6.2.1. NO Donors

NO donors (e.g., organic nitrates) require enzymatic activation, which is subject to tolerance, particularly in oxidative and inflammatory diseases [34]. The effect of NO donors may be limited due to the scavenging effect of increased levels of oxygen radicals that arise in blood vessels under inflammatory conditions [34]. The chronic administration of organic nitrates has been demonstrated to result in endothelial dysfunction [34,294]. The resulting ROS or RNS have been shown to induce the oxidation and dissociation of heme in NO-GC by oxidizing NO-GC, which can then no longer be activated by NO [33,220]. Furthermore, tolerance to organic nitrates has been observed to develop over time [34,229]. Therapeutic interventions for the NO-cGMP signaling cascade have thus focused on the identification of pharmacological agents that can directly activate the NO-sensitive enzyme NO-GC.

#### 6.2.2. The S-Glutathionylation of eNOS

The S-glutathionylation of eNOS is a reversible process [207,295]. This post-translational modification of eNOS could act as a regulatory mechanism to prevent the irreversible oxidation of cell components and reduce the formation of O_2_^−^ and ONOO^−^ under inflammatory conditions. Thus, the reversible S-glutathionylation of eNOS provides a unique redox mechanism by which the activity of eNOS may be regulated also in inflamed dental pulp.

#### 6.2.3. BH4 Supplementation

Under inflammatory conditions, BH4 is oxidized by ONOO^−^ to BH2, leading to the uncoupling of eNOS [32,196,198,201,296,297]. Therefore, BH4 is a potential therapeutic target for treating endothelial dysfunction under inflammatory conditions [49,50,51,196,198]. In animal models and in patients, supplementation with BH4 has been shown to correct endothelial dysfunction. In addition, folic acid and infusions of vitamin C are able to restore endothelial dysfunction enhancing BH4 levels [50]. The combination of BH4 supplementation and antioxidants is therefore recommended as a therapeutic approach to treat endothelial dysfunction [50,196,198,298]. However, the therapeutic potential of BH4 supplementation remains controversial [203]. In cases of inflammation, the effectiveness of oral BH4 supplementation is limited due to the rapid oxidation of BH4 to BH2 [299]. Furthermore, as iNOS is permanently active under inflammatory conditions, the supplementation of BH4 enhances iNOS activity, resulting in increased formation of ONOO^−^ in inflamed blood vessels [300].

### 6.3. NO-GC as a Therapeutic Target in Endothelial Dysfunction of Inflamed Dental Pulp

**Figure 5:**
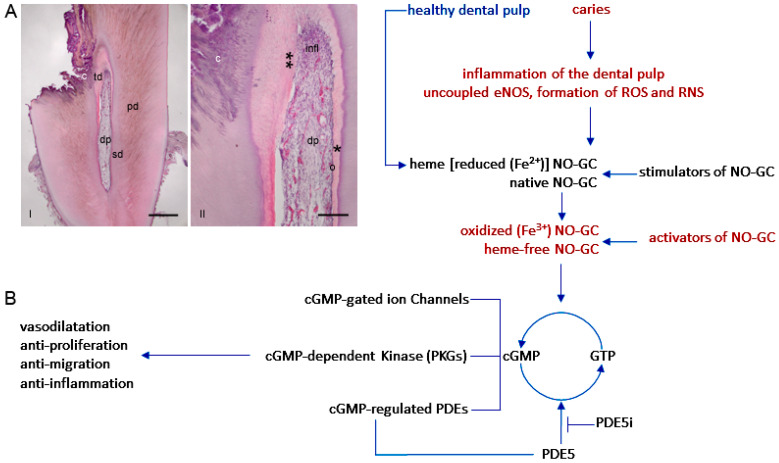
A deep carious lesion in a human premolar and a proposed caries treatment using NO-GC-stimulators and NO-GC-activators in dentin wound or pulp capping materials during caries treatment. (**A**) The overview image (**I**) shows deep dentin caries (c), inflamed pulp (dp), and tertiary dentin (td) formed in response to the carious lesion. The overview image (**I**) also shows areas of primary dentin (pd) and secondary dentin (sd). The detailed image (**II**) shows deep caries lesions (c), inflammatory cells (infl), dilated blood vessels, and the odontoblast layer (o) in the dental pulp. Reactive tertiary dentin matrix (one asterisk) and reparative tertiary dentin matrix (two asterisks) formed in response to the carious lesion are also visible. (**B**) A proposed schematic representation of the regulation of NO-GC activity in the blood vessels of the dental pulp by NO-GC stimulators, NO-GC activators, and the inhibition of cGMP degradation by PDE5i. The carious lesions induce an inflammatory response in the dental pulp. ROS and RNS are generated in higher concentrations in the inflamed pulp. In the inflamed dental pulp, eNOS is uncoupled. The uncoupled eNOS produces O_2_^−^ instead of NO. In inflamed vascular smooth muscle cells, the NO-GCβ-subunit contains a heme group that can exist either in an oxidized Fe^3+^ state or in a heme-free NO-GC state. NO-GC stimulators exhibit a dual mode of action, directly stimulating the native form of the enzyme and rendering it more sensitive to endogenous NO. The NO-GC activators have been shown to selectively activate dysfunctional, oxidized, and heme-free NO-GC. The stimulation of native NO-GC (Apo-NO-GC) by NO-GC-stimulators and the activation of heme-free NO-GC by NO-GC-activators have been shown to result in an increased formation of cGMP, which has been demonstrated to exert a significant, multifaceted, tissue-protective effect [302,309]. PDE5, which catalyzes the degradation of cGMP to GTP, is activated by ROS and RNS. The additional inhibition of cGMP degradation by PDE5 inhibitors has the potential to enhance NO-GC/cGMP signaling in vascular smooth muscle cells in inflamed dental pulp, exerting vasodilation, anti-proliferation, anti-migration and anti-inflammatory effects. Scale bar: (**I**) = 1 mm; (**II**) = 200 µm.

The binding of NO to the heme group of NO-GC catalyses the synthesis of cGMP, which in turn leads to vasodilation via a series of downstream mechanisms and inhibits the proliferation of VSMCs, platelet aggregation and leukocyte recruitment [18,301]. Impaired NO-cGMP signal transduction in the blood vessels of the inflamed dental pulp can lead to endothelial dysfunction and exacerbate proinflammatory processes. The oxidation of Fe^2+^ to Fe^3+^ in the heme group of NO-GC by ONOO^−^ desensitizes NO-GC to NO. In such instances, the activation of NO-cGMP by NO in the inflamed dental pulp becomes unfeasible. The loss of NO function in the endothelium and the impairment of NO-GC function in VSMCs under inflammatory conditions represent a pharmacological intervention target that can be achieved directly by activating NO-GC without NO [35,302]. In order to counteract the deleterious effects of NO-based treatments (e.g., organic nitrates) associated with inflammation, NO-GC activators could be used in dentin wounds and pulp capping materials to increase the synthesis of cGMP in the blood vessels of the inflamed dental pulp. In addition, identifying cellular factors that modulate NO-GC activity could lead to the development of new therapeutic strategies and improve the efficacy of existing NO-GC-targeted drugs [303] (Figure 5).

#### 6.3.1. NO-GC Stimulators and Activators

NO/NO-GC/cGMP signaling regulates VSMCs, fibroblasts, cardiomyocytes, muscle fibers, platelets, neurons and immune cells [36,119]. Thus, NO/NO-GC/cGMP signaling is involved in cell-specific regulation of processes such as vasodilation [17,18], fibrosis [35,304,305], neurotransmission [123,134], platelet aggregation [22,23], and inflammation [27,50]. The NO-GC stimulators and NO-GC activators target the NO/NO-GC/cGMP signaling by stimulating the heme-containing NO-GC and the heme-free NO-GC, respectively. This process triggers the formation of cGMP, which in turn mediates the beneficial effects in cells and tissues [33,109,302,306].

The usual endogenous oxidants of NO-GC include O_2_^−^ and ONOO^−^, which have been detected in inflamed dental pulp [12,234]. Consequently, NO-GC can be oxidized within the blood vessels of inflamed dental pulp. NO-GC stimulators and activators may exert their effectiveness in cases of inflammation of the dental pulp by restoring or improving NO/NO-GC/cGMP functions in the blood vessels (Figure 5).

##### NO-GC Stimulators

NO-GC stimulators target the redox state of NO-GC, in which the enzyme is NO-sensitive and reduced. NO-GC stimulators directly stimulate the native form of the enzyme independently of NO [36,307,308,309]. The action of NO-GC stimulators at the heme-containing enzyme is independent of NO but is enhanced in the presence of NO [36,109,302,308]. At the same time, NO-GC stimulators stabilise the nitrosyl heme complex of reduced NO-GC and therefore exhibit strong synergy with NO. NO-GC stimulators improve the responsiveness of NO-GC to low NO concentrations by stabilising its binding to NO [33,36,302] (Figure 5).

##### NO-GC Activators

NO-GC activators target the redox state of NO-GC, in which the enzyme is NO-insensitive and oxidized [33,109,220,223]. The presence of ROS and RNS can shift the redox balance of NO-GC in VSMCs to an oxidized, Fe^3+^-containing state, resulting in NO-GC developing a heme deficiency or becoming heme-free [33,222]. NO-GC activators bind to the unoccupied heme-binding complex or displace the prosthetic heme of NO-GC [36,302]. In certain cases, NO-GC activators have also been observed to protect the enzyme from degradation by proteasomes [33,302].

### 6.4. Phosphodiesterase-5 Inhibitors

Phosphodiesterases (PDEs) are ubiquitously expressed hydrolases that regulate the intracellular levels of cyclic nucleotides by hydrolysing cAMP to 5′AMP and cGMP to 5′GMP. To date, twenty-one human genes encoding phosphodiesterases (PDEs) have been identified. Based on their sequence homology, enzymatic properties and sensitivity to inhibitors, PDEs are classified into 11 distinct families [310,311,312]. In accordance with their activity, PDEs are divided into three groups. PDE4, PDE7 and PDE8, which belong to group I, have been shown to specifically hydrolyse only cAMP. PDE5, PDE6 and PDE9, which belong to group II, are responsible for the specific hydrolysis of only cGMP. PDE1, PDE2, PDE3, PDE10 and PDE11, which belong to group III, have the capacity to hydrolyse both cAMP and cGMP [310,311,312].

PDE5 has been demonstrated to be particularly effective in the degradation of cGMP [313,314], with evidence indicating that the inhibition of PDE5 can result in vasodilation [314,315]. The blood vessels of the dental pulp contain a significant concentration of cGMP [122]. Since inflammation of the dental pulp leads to the formation of ROS and RNS [12,234], and the activity of phosphodiesterases is increased by ROS and RNS [32,218], PDE5 inhibitors can be used pharmacologically to achieve an anti-inflammatory effect in the blood vessels of the inflamed dental pulp. Using PDE5 inhibitors in pulp capping materials during caries treatment has the potential to inhibit cGMP degradation in blood vessels of the dental pulp. This could improve NO-cGMP-dependent signaling in blood vessels of the inflamed dental pulp including vasodilation, the inhibition of platelet aggregation and leukocyte adhesion, and the inhibition of the proliferation and migration of VSMCs, exerting an anti-inflammatory effect (Figure 5).

## 7. Conclusions

**Figure 6:**
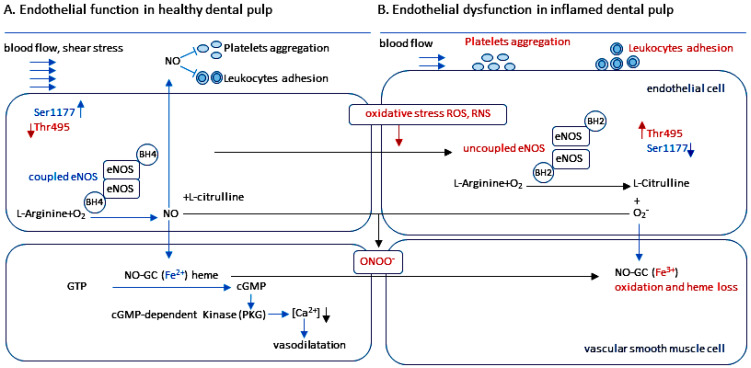
Endothelial function in healthy and inflamed dental pulp. (**A**) Endothelial function in healthy dental pulp. In the endothelial cells of healthy dental pulp, nitric oxide (NO) and L-citrulline are produced from L-arginine by endothelial nitric oxide synthase (eNOS), using existing cofactors such as reduced tetrahydrobiopterin (BH4). In comparison with other tissues, the relatively high intrapulpal tissue pressure in the dental pulp under physiological conditions can result in increased shear stress in endothelial cells [10]. This can trigger increased phosphorylation of eNOS at Ser1177 and weak phosphorylation of the enzyme at Thr495 in healthy dental pulp [12]. After formation, NO diffuses to underlying smooth muscle cells, binding to reduced heme (Fe^2+^) in nitric oxide-sensitive guanylyl cyclase (NO-GC) to activate the enzyme. This leads to the formation of cyclic guanosine 3′,5′-monophosphate (cGMP) from guanosine 5’-triphosphate (GTP). The downstream signaling cascade is activated by cGMP-dependent protein kinase (PKG), which leads to a reduction in Ca^2+^ concentrations in the smooth muscle cells, resulting in their relaxation. NO production in the endothelial cells of healthy dental pulp also prevents the expression of endothelial cell adhesion molecules and chemokines, thereby preventing platelet activation, aggregation, and leukocyte adhesion. (**B**) Endothelial dysfunction in inflamed dental pulp. In inflamed dental pulp following a carious lesion, ROS and RNS are produced at higher concentrations. ROS and RNS cause the uncoupling of eNOS by oxidizing the cofactor BH4 to dihydrobiopterin (BH2). Uncoupled eNOS then forms superoxide (O_2_^−^) instead of NO. Under inflammatory conditions, eNOS phosphorylation at Thr495 increases and at Ser1177 decreases. This can additionally reduce eNOS activity and lead to the formation of O_2_^−^ instead of NO. Under inflammatory conditions in the dental pulp, the bioavailability of NO is thus severely restricted. The formed O_2_^−^ reacts with NO to form peroxynitrite (ONOO^−^). ONOO^−^ then oxidizes the heme group in NO-GC from Fe^2+^ to Fe^3+^. The oxidized NO-GC is then insensitive to NO. The formation of ROS and RNS, as well as the proinflammatory activation of the endothelium, leads to the expression of endothelial cell adhesion molecules and chemokines. This results in platelet activation and aggregation, as well as leukocyte adhesion, in the blood vessels of the inflamed dental pulp.

In the context of carious lesions, endothelial dysfunction develops due to reduced endogenous NO levels and impaired NO-cGMP signaling in the blood vessels of the inflamed dental pulp. Endothelial dysfunction in inflamed dental pulp can be caused by a number of factors, including a reduction in the bioavailability of L-arginine, the uncoupling of eNOS through the oxidation of BH4 to BH2, the inactivation of endogenous NO by O_2_^−^ forming ONOO^−^, increased cellular concentrations of the eNOS inhibitor ADMA, and ROS- and RNS-oxidized, heme-free and thus NO-insensitive NO-GC. In the blood vessels of the inflamed dental pulp, a number of PDEs, particularly PDE5, are activated by ROS and RNS to regulate the enzymatic cleavage of cGMP (Figure 6). The results of research in the field of NO-cGMP pharmacology in the cardiovascular system could also provide a scientific basis for the treatment of endothelial dysfunction in blood vessels of the inflamed dental pulp. To treat endothelial dysfunction of the inflamed dental pulp, NO donors, PDE inhibitors, NO-GC stimulators, and NO-GC activators may be used in indirect and direct pulp capping materials.

## 8. Perspective

The dental pulp is located within a rigid dentin chamber and is only accessible to blood vessels and nerve fibers through the apical foramen. This unique structure exhibits intrapulpal tissue pressure, which can be altered under physiological and inflammatory conditions. Changes in intrapulpal tissue pressure can affect the production of NO in endothelial cells, which influences the activity of the NO-GC/cGMP signaling cascade in vascular smooth muscle cells in blood vessels of the dental pulp. Due to complicated accessibility, studies on endothelial function and dysfunction in the dental pulp are limited. In this review, we present evidence from our studies and those of other researchers showing that NO/NO-GC/cGMP signaling in the dental pulp plays an important role in endothelial function and dysfunction. The formation of ROS and RNS in an inflamed dental pulp should be considered a trigger for endothelial dysfunction. Further studies are needed to characterize the formation of ROS and RNS in inflamed dental pulp. These compounds could therefore represent an attractive therapeutic target for treating caries in inflamed dental pulp.

## Data Availability

No new data were created or analyzed in this study.

## References

[1] Takahashi K. (1985). Vascular architecture of dog pulp using corrosion resin cast examined under a scanning electron microscope. J. Dent. Res..

[2] Kim S. (1985). Microcirculation of the dental pulp in health and disease. J. Endod..

[3] Yoshida S., Ohshima H. (1996). Distribution and organization of peripheral capillaries in dental pulp and their relationship to odontoblasts. Anat. Rec..

[4] Smith A.J. (2002). Pulpal responses to caries and dental repair. Caries Res..

[5] Cooper P.R., Holder M.J., Smith A.J. (2014). Inflammation and regeneration in the dentin-pulp complex: A double-edged sword. J. Endod..

[6] Farges J.C., Alliot-Licht B., Renard E., Ducret M., Gaudin A., Smith A.J., Cooper P.R. (2015). Dental Pulp Defence and Repair Mechanisms in Dental Caries. Mediat. Inflamm..

[7] Smith A.J., Duncan H.F., Diogenes A., Simon S., Cooper P.R. (2016). Exploiting the Bioactive Properties of the Dentin-Pulp Complex in Regenerative Endodontics. J. Endod..

[8] Kim S. (1990). Neurovascular interactions in the dental pulp in health and inflammation. J. Endod..

[9] Heyeraas K.J., Kvinnsland I. (1992). Tissue pressure and blood flow in pulpal inflammation. Proc. Finn. Dent. Soc..

[10] Heyeraas K.J., Berggreen E. (1999). Interstitial fluid pressure in normal and inflamed pulp. Crit. Rev. Oral Biol. Med..

[11] Berggreen E., Bletsa A., Heyeraas K.J. (2007). Circulation in normal and inflamed dental pulp. Endod. Top..

[12] Erdek Ö., Bloch W., Rink-Notzon S., Roggendorf H.C., Uzun S., Meul B., Koch M., Neugebauer J., Deschner J., Korkmaz Y. (2022). Inflammation of the Human Dental Pulp Induces Phosphorylation of eNOS at Thr495 in Blood Vessels. Biomedicines.

[13] Vanhoutte P.M., Zhao Y., Xu A., Leung S.W. (2016). Thirty Years of Saying NO: Sources, Fate, Actions, and Misfortunes of the Endothelium-Derived Vasodilator Mediator. Circ. Res..

[14] Gutterman D.D., Chabowski D.S., Kadlec A.O., Durand M.J., Freed J.K., Ait-Aissa K., Beyer A.M. (2016). The Human Microcirculation: Regulation of Flow and Beyond. Circ. Res..

[15] Oates J.C., Russell D.L., Van Beusecum J.P. (2022). Endothelial cells: Potential novel regulators of renal inflammation. Am. J. Physiol. Ren. Physiol..

[16] Hall C.N., Garthwaite J. (2009). What is the real physiological NO concentration in vivo?. Nitric Oxide.

[17] Friebe A., Koesling D. (2003). Regulation of nitric oxide-sensitive guanylyl cyclase. Circ. Res..

[18] Murad, Shattuck Lecture F. (2006). Nitric oxide and cyclic GMP in cell signaling and drug development. N. Engl. J. Med..

[19] Moncada S., Higgs A. (1993). The L-arginine-nitric oxide pathway. N. Engl. J. Med..

[20] Loscalzo J. (2001). Nitric oxide insufficiency, platelet activation, and arterial thrombosis. Circ. Res..

[21] Dangel O., Mergia E., Karlisch K., Groneberg D., Koesling D., Friebe A. (2010). Nitric oxide-sensitive guanylyl cyclase is the only nitric oxide receptor mediating platelet inhibition. J. Thromb. Haemost..

[22] Makhoul S., Walter E., Pagel O., Walter U., Sickmann A., Gambaryan S., Smolenski A., Zahedi R.P., Jurk K. (2018). Effects of the NO/soluble guanylate cyclase/cGMP system on the functions of human platelets. Nitric Oxide.

[23] Mauersberger C., Sager H.B., Wobst J., Dang T.A., Lambrecht L., Koplev S., Stroth M., Bettaga N., Schlossmann J., Wunder F. (2022). Loss of soluble guanylyl cyclase in platelets contributes to atherosclerotic plaque formation and vascular inflammation. Nat. Cardiovasc. Res..

[24] Sneddon J.M., Vane J.R. (1988). Endothelium-derived relaxing factor reduces platelet adhesion to bovine endothelial cells. Proc. Natl. Acad. Sci. USA.

[25] Radomski M.W., Palmer R.M., Moncada S. (1990). An L-arginine/nitric oxide pathway present in human platelets regulates aggregation. Proc. Natl. Acad. Sci. USA.

[26] Ahluwalia A., Foster P., Scotland R.S., McLean P.G., Mathur A., Perretti M., Moncada S., Hobbs A.J. (2004). Antiinflammatory activity of soluble guanylate cyclase: cGMP-dependent down-regulation of P-selectin expression and leukocyte recruitment. Proc. Natl. Acad. Sci. USA.

[27] Gimbrone M.A., García-Cardeña G. (2016). Endothelial Cell Dysfunction and the Pathobiology of Atherosclerosis. Circ. Res..

[28] Garg U.C., Hassid A. (1989). Nitric oxide-generating vasodilators and 8-bromo-cyclic guanosine monophosphate inhibit mitogenesis and proliferation of cultured rat vascular smooth muscle cells. J. Clin. Investig..

[29] Rudic R.D., Shesely E.G., Maeda N., Smithies O., Segal S.S., Sessa W.C. (1998). Direct evidence for the importance of endothelium-derived nitric oxide in vascular remodeling. J. Clin. Investig..

[30] Napoli C., Paolisso G., Casamassimi A., Al-Omran M., Barbieri M., Sommese L., Infante T., Ignarro L.J. (2013). Effects of nitric oxide on cell proliferation: Novel insights. J. Am. Coll. Cardiol..

[31] Hildebrand S., Ibrahim M., Schlitzer A., Maegdefessel L., Röll W., Pfeifer A. (2022). PDGF regulates guanylate cyclase expression and cGMP signaling in vascular smooth muscle. Commun. Biol..

[32] Münzel T., Daiber A., Ullrich V., Mülsch A. (2005). Vascular consequences of endothelial nitric oxide synthase uncoupling for the activity and expression of the soluble guanylyl cyclase and the cGMP-dependent protein kinase. Arterioscler. Thromb. Vasc. Biol..

[33] Stasch J.P., Pacher P., Evgenov O.V. (2011). Soluble guanylate cyclase as an emerging therapeutic target in cardiopulmonary disease. Circulation.

[34] Lundberg J.O., Gladwin M.T., Weitzberg E. (2015). Strategies to increase nitric oxide signalling in cardiovascular disease. Nat. Rev. Drug Discov..

[35] Sandner P., Stasch J.P. (2017). Anti-fibrotic effects of soluble guanylate cyclase stimulators and activators: A review of the preclinical evidence. Respir. Med..

[36] Sandner P., Zimmer D.P., Milne G.T., Follmann M., Hobbs A., Stasch J.P. (2021). Soluble Guanylate Cyclase Stimulators and Activators. Handb. Exp. Pharmacol..

[37] Daiber A., Chlopicki S. (2020). Revisiting pharmacology of oxidative stress and endothelial dysfunction in cardiovascular disease: Evidence for redox-based therapies. Free Radic. Biol. Med..

[38] Kuhn M. (2003). Structure, regulation, and function of mammalian membrane guanylyl cyclase receptors, with a focus on guanylyl cyclase-A. Circ. Res..

[39] Kuhn M. (2016). Molecular Physiology of Membrane Guanylyl Cyclase Receptors. Physiol. Rev..

[40] Castro L.R., Verde I., Cooper D.M., Fischmeister R. (2006). Cyclic guanosine monophosphate compartmentation in rat cardiac myocytes. Circulation.

[41] Takimoto E., Belardi D., Tocchetti C.G., Vahebi S., Cormaci G., Ketner E.A., Moens A.L., Champion H.C., Kass D.A. (2007). Compartmentalization of cardiac β-adrenergic inotropy modulation by phosphodiesterase type 5. Circulation.

[42] Lim S.L., Lam C.S., Segers V.F., Brutsaert D.L., De Keulenaer G.W. (2015). Cardiac endothelium-myocyte interaction: Clinical opportunities for new heart failure therapies regardless of ejection fraction. Eur. Heart J..

[43] Förstermann U., Closs E.I., Pollock J.S., Nakane M., Schwarz P., Gath I., Kleinert H. (1994). Nitric oxide synthase isozymes. Characterization, purification, molecular cloning, and functions. Hypertension.

[44] Stuehr D.J. (1999). Mammalian nitric oxide synthases. Biochim. Biophys. Acta.

[45] Alderton W.K., Cooper C.E., Knowles R.G. (2001). Nitric oxide synthases: Structure, function and inhibition. Biochem. J..

[46] Daff S. (2010). NO synthase: Structures and mechanisms. Nitric Oxide.

[47] Förstermann U., Sessa W.C. (2012). Nitric oxide synthases: Regulation and function. Eur. Heart J..

[48] Fleming I. (2010). Molecular mechanisms underlying the activation of eNOS. Pflug. Arch..

[49] Tejero J., Shiva S., Gladwin M.T. (2019). Sources of Vascular Nitric Oxide and Reactive Oxygen Species and Their Regulation. Physiol. Rev..

[50] Förstermann U., Münzel T. (2006). Endothelial nitric oxide synthase in vascular disease: From marvel to menace. Circulation.

[51] Li H., Forstermann U. (2014). Pharmacological prevention of eNOS uncoupling. Curr. Pharm. Des..

[52] Mishra S., Chander V., Kass D.A. (2025). Cardiac cGMP Regulation and Therapeutic Applications. Hypertension.

[53] Farah C., Michel L.Y.M., Balligand J.L. (2018). Nitric oxide signalling in cardiovascular health and disease. Nat. Rev. Cardiol..

[54] Feron O., Michel J.B., Sase K., Michel T. (1998). Dynamic regulation of endothelial nitric oxide synthase: Complementary roles of dual acylation and caveolin interactions. Biochemistry.

[55] Belhassen L., Feron O., Kaye D.M., Michel T., Kelly R.A. (1997). Regulation by cAMP of post-translational processing and subcellular targeting of endothelial nitric-oxide synthase (type 3) in cardiac myocytes. J. Biol. Chem..

[56] García-Cardeña G., Oh P., Liu J., Schnitzer J.E., Sessa W.C. (1996). Targeting of nitric oxide synthase to endothelial cell caveolae via palmitoylation: Implications for nitric oxide signaling. Proc. Natl. Acad. Sci. USA.

[57] Boissel J.-P., Schwarz P.M., Förstermann U. (1998). Neuronal-Type NO Synthase: Transcript Diversity and Expressional Regulation. Nitric Oxide.

[58] Zhou L., Zhu D.Y. (2009). Neuronal nitric oxide synthase: Structure, subcellular localization, regulation, and clinical implications. Nitric Oxide.

[59] Bredt D.S., Hwang P.M., Snyder S.H. (1990). Localization of nitric oxide synthase indicating a neural role for nitric oxide. Nature.

[60] Bredt D.S., Glatt C.E., Hwang P.M., Fotuhi M., Dawson T.M., Snyder S.H. (1991). Nitric oxide synthase protein and mRNA are discretely localized in neuronal populations of the mammalian CNS together with NADPH diaphorase. Neuron.

[61] Rand M.J., Li C.G. (1995). Nitric oxide as a neurotransmitter in peripheral nerves: Nature of transmitter and mechanism of transmission. Annu. Rev. Physiol..

[62] Hedlund P., Alm P., Andersson K.E. (1999). NO synthase in cholinergic nerves and NO-induced relaxation in the rat isolated corpus cavernosum. Br. J. Pharmacol..

[63] Boulanger C.M., Heymes C., Benessiano J., Geske R.S., Levy B.I., Vanhoutte P.M. (1998). Neuronal nitric oxide synthase is expressed in rat vascular smooth muscle cells: Activation by angiotensin II in hypertension. Circ. Res..

[64] Schwarz P.M., Kleinert H., Förstermann U. (1999). Potential functional significance of brain-type and muscle-type nitric oxide synthase I expressed in adventitia and media of rat aorta. Arterioscler. Thromb. Vasc. Biol..

[65] Nakane M., Schmidt H.H., Pollock J.S., Förstermann U., Murad F. (1993). Cloned human brain nitric oxide synthase is highly expressed in skeletal muscle. FEBS Lett..

[66] Kobzik L., Reid M.B., Bredt D.S., Stamler J.S. (1994). Nitric oxide in skeletal muscle. Nature.

[67] Stamler J.S., Meissner G. (2001). Physiology of nitric oxide in skeletal muscle. Physiol. Rev..

[68] Rothe F., Langnaese K., Wolf G. (2005). New aspects of the location of neuronal nitric oxide synthase in the skeletal muscle: A light and electron microscopic study. Nitric Oxide.

[69] Izumi Y., Clifford D.B., Zorumski C.F. (1992). Inhibition of long-term potentiation by NMDA-mediated nitric oxide release. Science.

[70] Izumi Y., Zorumski C.F. (1993). Nitric oxide and long-term synaptic depression in the rat hippocampus. Neuroreport.

[71] Bredt D.S., Snyder S.H. (1992). Nitric oxide, a novel neuronal messenger. Neuron.

[72] Calabrese V., Mancuso C., Calvani M., Rizzarelli E., Butterfield D.A., Stella A.M. (2007). Nitric oxide in the central nervous system: Neuroprotection versus neurotoxicity. Nat. Rev. Neurosci..

[73] Garthwaite J. (2008). Concepts of neural nitric oxide-mediated transmission. Eur. J. Neurosci..

[74] Bredt D.S. (1999). Endogenous nitric oxide synthesis: Biological functions and pathophysiology. Free Radic. Res..

[75] Melikian N., Seddon M.D., Casadei B., Chowienczyk P.J., Shah A.M. (2009). Neuronal nitric oxide synthase and human vascular regulation. Trends Cardiovasc. Med..

[76] Kim N., Azadzoi K.M., Goldstein I., Saenz de Tejada I. (1991). A nitric oxide-like factor mediates nonadrenergic-noncholinergic neurogenic relaxation of penile corpus cavernosum smooth muscle. J. Clin. Investig..

[77] Rajfer J., Aronson W.J., Bush P.A., Dorey F.J., Ignarro L.J. (1992). Nitric oxide as a mediator of relaxation of the corpus cavernosum in response to nonadrenergic, noncholinergic neurotransmission. N. Engl. J. Med..

[78] Toda N., Okamura T. (2003). The pharmacology of nitric oxide in the peripheral nervous system of blood vessels. Pharmacol. Rev..

[79] Toda N., Ayajiki K., Okamura T. (2005). Nitric oxide and penile erectile function. Pharmacol. Ther..

[80] Marsden P.A., Heng H.H., Scherer S.W., Stewart R.J., Hall A.V., Shi X.M., Tsui L.C., Schappert K.T. (1993). Structure and chromosomal localization of the human constitutive endothelial nitric oxide synthase gene. J. Biol. Chem..

[81] Ignarro L.J., Byrns R.E., Buga G.M., Wood K.S. (1987). Endothelium-derived relaxing factor from pulmonary artery and vein possesses pharmacologic and chemical properties identical to those of nitric oxide radical. Circ. Res..

[82] Palmer R.M., Ferrige A.G., Moncada S. (1987). Nitric oxide release accounts for the biological activity of endothelium-derived relaxing factor. Nature.

[83] Moncada S., Palmer R.M., Higgs E.A. (1988). The discovery of nitric oxide as the endogenous nitrovasodilator. Hypertension.

[84] Forstermann U., Pollock J.S., Schmidt H.H., Heller M., Murad F. (1991). Calmodulin-dependent endothelium-derived relaxing factor/nitric oxide synthase activity is present in the particulate and cytosolic fractions of bovine aortic endothelial cells. Proc. Natl. Acad. Sci. USA.

[85] Rapoport R.M., Draznin M.B., Murad F. (1983). Endothelium-dependent relaxation in rat aorta may be mediated through cyclic GMP-dependent protein phosphorylation. Nature.

[86] Furchgott R.F., Cherry P.D., Zawadzki J.V., Jothianandan D. (1984). Endothelial cells as mediators of vasodilation of arteries. J. Cardiovasc. Pharmacol..

[87] Förstermann U., Mülsch A., Böhme E., Busse R. (1986). Stimulation of soluble guanylate cyclase by an acetylcholine-induced endothelium-derived factor from rabbit and canine arteries. Circ. Res..

[88] Murohara T., Witzenbichler B., Spyridopoulos I., Asahara T., Ding B., Sullivan A., Losordo D.W., Isner J.M. (1999). Role of endothelial nitric oxide synthase in endothelial cell migration. Arterioscler. Thromb. Vasc. Biol..

[89] Karbach S., Wenzel P., Waisman A., Munzel T., Daiber A. (2014). eNOS uncoupling in cardiovascular diseases—The role of oxidative stress and inflammation. Curr. Pharm. Des..

[90] Daiber A., Steven S., Weber A., Shuvaev V.V., Muzykantov V.R., Laher I., Li H., Lamas S., Münzel T. (2017). Targeting vascular (endothelial) dysfunction. Br. J. Pharmacol..

[91] Stuehr D.J., Cho H.J., Kwon N.S., Weise M.F., Nathan C.F. (1991). Purification and characterization of the cytokine-induced macrophage nitric oxide synthase: An FAD- and FMN-containing flavoprotein. Proc. Natl. Acad. Sci. USA.

[92] Nathan C., Xie Q.W. (1994). Nitric oxide synthases: Roles, tolls, and controls. Cell.

[93] Xie Q.W., Kashiwabara Y., Nathan C. (1994). Role of transcription factor NF-kappa B/Rel in induction of nitric oxide synthase. J. Biol. Chem..

[94] de Vera M.E., Shapiro R.A., Nussler A.K., Mudgett J.S., Simmons R.L., Morris S.M., Billiar T.R., Geller D.A. (1996). Transcriptional regulation of human inducible nitric oxide synthase (NOS2) gene by cytokines: Initial analysis of the human NOS2 promoter. Proc. Natl. Acad. Sci. USA.

[95] Pautz A., Art J., Hahn S., Nowag S., Voss C., Kleinert H. (2010). Regulation of the expression of inducible nitric oxide synthase. Nitric Oxide.

[96] Balligand J.L., Ungureanu-Longrois D., Simmons W.W., Pimental D., Malinski T.A., Kapturczak M., Taha Z., Lowenstein C.J., Davidoff A.J., Kelly R.A. (1994). Cytokine-inducible nitric oxide synthase (iNOS) expression in cardiac myocytes. Characterization and regulation of iNOS expression and detection of iNOS activity in single cardiac myocytes in vitro. J. Biol. Chem..

[97] Haywood G.A., Tsao P.S., von der Leyen H.E., Mann M.J., Keeling P.J., Trindade P.T., Lewis N.P., Byrne C.D., Rickenbacher P.R., Bishopric N.H. (1996). Expression of inducible nitric oxide synthase in human heart failure. Circulation.

[98] Wilcox J.N., Subramanian R.R., Sundell C.L., Tracey W.R., Pollock J.S., Harrison D.G., Marsden P.A. (1997). Expression of multiple isoforms of nitric oxide synthase in normal and atherosclerotic vessels. Arterioscler. Thromb. Vasc. Biol..

[99] MacMicking J., Xie Q.W., Nathan C. (1997). Nitric oxide and macrophage function. Annu. Rev. Immunol..

[100] Xia Y., Zweier J.L. (1997). Superoxide and peroxynitrite generation from inducible nitric oxide synthase in macrophages. Proc. Natl. Acad. Sci. USA.

[101] Cinelli M.A., Do H.T., Miley G.P., Silverman R.B. (2020). Inducible nitric oxide synthase: Regulation, structure, and inhibition. Med. Res. Rev..

[102] Billaud M., Lohman A.W., Johnstone S.R., Biwer L.A., Mutchler S., Isakson B.E. (2014). Regulation of cellular communication by signaling microdomains in the blood vessel wall. Pharmacol. Rev..

[103] Frank P.G., Woodman S.E., Park D.S., Lisanti M.P. (2003). Caveolin, caveolae, and endothelial cell function. Arterioscler. Thromb. Vasc. Biol..

[104] Gratton J.P., Bernatchez P., Sessa W.C. (2004). Caveolae and caveolins in the cardiovascular system. Circ. Res..

[105] Fontana J., Fulton D., Chen Y., Fairchild T.A., McCabe T.J., Fujita N., Tsuruo T., Sessa W.C. (2002). Domain mapping studies reveal that the M domain of hsp90 serves as a molecular scaffold to regulate Akt-dependent phosphorylation of endothelial nitric oxide synthase and NO release. Circ. Res..

[106] Balligand J.L., Feron O., Dessy C. (2009). eNOS activation by physical forces: From short-term regulation of contraction to chronic remodeling of cardiovascular tissues. Physiol. Rev..

[107] Dimmeler S., Fleming I., Fisslthaler B., Hermann C., Busse R., Zeiher A.M. (1999). Activation of nitric oxide synthase in endothelial cells by Akt-dependent phosphorylation. Nature.

[108] Fleming I., Busse R. (2003). Molecular mechanisms involved in the regulation of the endothelial nitric oxide synthase. Am. J. Physiol. Regul. Integr. Comp. Physiol..

[109] Evgenov O.V., Pacher P., Schmidt P.M., Haskó G., Schmidt H.H., Stasch J.P. (2006). NO-independent stimulators and activators of soluble guanylate cyclase: Discovery and therapeutic potential. Nat. Rev. Drug Discov..

[110] Friebe A., Koesling D. (2009). The function of NO-sensitive guanylyl cyclase: What we can learn from genetic mouse models. Nitric Oxide.

[111] Shah R.C., Sanker S., Wood K.C., Durgin B.G., Straub A.C. (2018). Redox regulation of soluble guanylyl cyclase. Nitric Oxide.

[112] Zabel U., Häusler C., Weeger M., Schmidt H.H. (1999). Homodimerization of soluble guanylyl cyclase subunits. Dimerization analysis using a glutathione s-transferase affinity tag. J. Biol. Chem..

[113] Fernhoff N.B., Derbyshire E.R., Marletta M.A. (2009). A nitric oxide/cysteine interaction mediates the activation of soluble guanylate cyclase. Proc. Natl. Acad. Sci. USA.

[114] Dent M.R., DeMartino A.W., Tejero J., Gladwin M.T. (2021). Endogenous Hemoprotein-Dependent Signaling Pathways of Nitric Oxide and Nitrite. Inorg. Chem..

[115] Sharina I.G., Krumenacker J.S., Martin E., Murad F. (2000). Genomic organization of α1 and β1 subunits of the mammalian soluble guanylyl cyclase genes. Proc. Natl. Acad. Sci. USA.

[116] Sharina I.G., Cote G.J., Martin E., Doursout M.F., Murad F. (2011). RNA splicing in regulation of nitric oxide receptor soluble guanylyl cyclase. Nitric Oxide.

[117] Gibb B.J., Garthwaite J. (2001). Subunits of the nitric oxide receptor, soluble guanylyl cyclase, expressed in rat brain. Eur. J. Neurosci..

[118] Bellamy T.C., Garthwaite J. (2002). Pharmacology of the nitric oxide receptor, soluble guanylyl cyclase, in cerebellar cells. Br. J. Pharmacol..

[119] Friebe A., Voußen B., Groneberg D. (2018). NO-GC in cells ‘off the beaten track’. Nitric Oxide.

[120] Korkmaz Y., Pryymachuk G., Schroeter M.M., Puladi B., Piekarek N., Appel S., Bloch W., Lackmann J.W., Deschner J., Friebe A. (2024). The α_1_- and β_1_-Subunits of Nitric Oxide-Sensitive Guanylyl Cyclase in Pericytes of Healthy Human Dental Pulp. Int. J. Mol. Sci..

[121] Mergia E., Russwurm M., Zoidl G., Koesling D. (2003). Major occurrence of the new α_2_β_1_ isoform of NO-sensitive guanylyl cyclase in brain. Cell. Signal..

[122] Korkmaz Y., Baumann M.A., Steinritz D., Schröder H., Behrends S., Addicks K., Schneider K., Raab W.H., Bloch W. (2005). NO-cGMP signaling molecules in cells of the rat molar dentin-pulp complex. J. Dent. Res..

[123] Koesling D., Mergia E., Russwurm M. (2016). Physiological Functions of NO-Sensitive Guanylyl Cyclase Isoforms. Curr. Med. Chem..

[124] Yuen P.S., Potter L.R., Garbers D.L. (1990). A new form of guanylyl cyclase is preferentially expressed in rat kidney. Biochemistry.

[125] Behrends S., Budaeus L., Kempfert J., Scholz H., Starbatty J., Vehse K. (2001). The β_2_ subunit of nitric oxide-sensitive guanylyl cyclase is developmentally regulated in rat kidney. Naunyn-Schmiedeberg’s Arch. Pharmacol..

[126] Gupta G., Azam M., Yang L., Danziger R.S. (1997). The β2 subunit inhibits stimulation of the α1/β1 form of soluble guanylyl cyclase by nitric oxide. Potential relevance to regulation of blood pressure. J. Clin. Investig..

[127] Nelissen E., Schepers M., Ponsaerts L., Foulquier S., Bronckaers A., Vanmierlo T., Sandner P., Prickaerts J. (2023). Soluble guanylyl cyclase: A novel target for the treatment of vascular cognitive impairment?. Pharmacol. Res..

[128] Lucas K.A., Pitari G.M., Kazerounian S., Ruiz-Stewart I., Park J., Schulz S., Chepenik K.P., Waldman S.A. (2000). Guanylyl cyclases and signaling by cyclic GMP. Pharmacol. Rev..

[129] Derbyshire E.R., Marletta M.A. (2012). Structure and regulation of soluble guanylate cyclase. Annu. Rev. Biochem..

[130] Beuve A. (2017). Thiol-Based Redox Modulation of Soluble Guanylyl Cyclase, the Nitric Oxide Receptor. Antioxid. Redox Signal..

[131] Kang Y., Liu R., Wu J.X., Chen L. (2019). Structural insights into the mechanism of human soluble guanylate cyclase. Nature.

[132] Montfort W.R., Wales J.A., Weichsel A. (2017). Structure and Activation of Soluble Guanylyl Cyclase, the Nitric Oxide Sensor. Antioxid. Redox Signal..

[133] Horst B.G., Marletta M.A. (2018). Physiological activation and deactivation of soluble guanylate cyclase. Nitric Oxide.

[134] Burette A., Zabel U., Weinberg R.J., Schmidt H.H., Valtschanoff J.G. (2002). Synaptic localization of nitric oxide synthase and soluble guanylyl cyclase in the hippocampus. J. Neurosci..

[135] Budworth J., Meillerais S., Charles I., Powell K. (1999). Tissue distribution of the human soluble guanylate cyclases. Biochem. Biophys. Res. Commun..

[136] Stuehr D.J., Biswas P., Dai Y., Ghosh A., Islam S., Jayaram D.T. (2023). A natural heme deficiency exists in biology that allows nitric oxide to control heme protein functions by regulating cellular heme distribution. Bioessays.

[137] Dai Y., Stuehr D.J. (2025). Heme delivery into soluble guanylyl cyclase requires a heme redox change and is regulated by NO and Hsp90 by distinct mechanisms. J. Biol. Chem..

[138] Dai Y., Faul E.M., Ghosh A., Stuehr D.J. (2022). NO rapidly mobilizes cellular heme to trigger assembly of its own receptor. Proc. Natl. Acad. Sci. USA.

[139] Karagöz G.E., Rüdiger S.G. (2015). Hsp90 interaction with clients. Trends Biochem. Sci..

[140] Ghosh A., Stasch J.P., Papapetropoulos A., Stuehr D.J. (2014). Nitric oxide and heat shock protein 90 activate soluble guanylate cyclase by driving rapid change in its subunit interactions and heme content. J. Biol. Chem..

[141] Dai Y., Schlanger S., Haque M.M., Misra S., Stuehr D.J. (2019). Heat shock protein 90 regulates soluble guanylyl cyclase maturation by a dual mechanism. J. Biol. Chem..

[142] Dai Y., Sweeny E.A., Schlanger S., Ghosh A., Stuehr D.J. (2020). GAPDH delivers heme to soluble guanylyl cyclase. J. Biol. Chem..

[143] Sweeny E.A., Schlanger S., Stuehr D.J. (2020). Dynamic regulation of NADPH oxidase 5 by intracellular heme levels and cellular chaperones. Redox Biol..

[144] Stuehr D.J., Misra S., Dai Y., Ghosh A. (2021). Maturation, inactivation, and recovery mechanisms of soluble guanylyl cyclase. J. Biol. Chem..

[145] Gao Y. (2010). The multiple actions of NO. Pflug. Arch..

[146] Arnold W.P., Mittal C.K., Katsuki S., Murad F. (1977). Nitric oxide activates guanylate cyclase and increases guanosine 3′:5′-cyclic monophosphate levels in various tissue preparations. Proc. Natl. Acad. Sci. USA.

[147] Waldman S.A., Murad F. (1987). Cyclic GMP synthesis and function. Pharmacol. Rev..

[148] Hofmann F. (2005). The biology of cyclic GMP-dependent protein kinases. J. Biol. Chem..

[149] Biel M., Sautter A., Ludwig A., Hofmann F., Zong X. (1998). Cyclic nucleotide-gated channels--mediators of NO:cGMP-regulated processes. Naunyn-Schmiedeberg’s Arch. Pharmacol..

[150] Fischmeister R., Castro L., Abi-Gerges A., Rochais F., Vandecasteele G. (2005). Species- and tissue-dependent effects of NO and cyclic GMP on cardiac ion channels. Comp. Biochem. Physiol. A Mol. Integr. Physiol..

[151] Rybalkin S.D., Yan C., Bornfeldt K.E., Beavo J.A. (2003). Cyclic GMP phosphodiesterases and regulation of smooth muscle function. Circ. Res..

[152] Landmesser U., Hornig B., Drexler H. (2004). Endothelial function: A critical determinant in atherosclerosis?. Circulation.

[153] Magloire H., Romeas A., Melin M., Couble M.L., Bleicher F., Farges J.C. (2001). Molecular regulation of odontoblast activity under dentin injury. Adv. Dent. Res..

[154] Bleicher F. (2014). Odontoblast physiology. Exp. Cell Res..

[155] Chmilewsky F., About I., Chung S.H. (2016). Pulp Fibroblasts Control Nerve Regeneration through Complement Activation. J. Dent. Res..

[156] Álvarez-Vásquez J.L., Castañeda-Alvarado C.P. (2022). Dental Pulp Fibroblast: A Star Cell. J. Endod..

[157] Byers M.R. (1984). Dental sensory receptors. Int. Rev. Neurobiol..

[158] Byers M.R., Närhi M.V. (1999). Dental injury models: Experimental tools for understanding neuroinflammatory interactions and polymodal nociceptor functions. Crit. Rev. Oral Biol. Med..

[159] Kaukua N., Shahidi M.K., Konstantinidou C., Dyachuk V., Kaucka M., Furlan A., An Z., Wang L., Hultman I., Ahrlund-Richter L. (2014). Glial origin of mesenchymal stem cells in a tooth model system. Nature.

[160] Iwasaki Y., Otsuka H., Yanagisawa N., Hisamitsu H., Manabe A., Nonaka N., Nakamura M. (2011). In situ proliferation and differentiation of macrophages in dental pulp. Cell Tissue Res..

[161] Yoshiba N., Edanami N., Ohkura N., Maekawa T., Takahashi N., Tohma A., Izumi K., Maeda T., Hosoya A., Nakamura H. (2020). M2 Phenotype Macrophages Colocalize with Schwann Cells in Human Dental Pulp. J. Dent. Res..

[162] Bhingare A.C., Ohno T., Tomura M., Zhang C., Aramaki O., Otsuki M., Tagami J., Azuma M. (2014). Dental pulp dendritic cells migrate to regional lymph nodes. J. Dent. Res..

[163] Korkmaz Y., Imhof T., Kämmerer P.W., Bloch W., Rink-Notzon S., Möst T., Weber M., Kesting M., Galler K.M., Deschner J. (2022). The colocalizations of pulp neural stem cells markers with dentin matrix protein-1, dentin sialoprotein and dentin phosphoprotein in human denticle (pulp stone) lining cells. Ann. Anat..

[164] Gronthos S., Mankani M., Brahim J., Robey P.G., Shi S. (2000). Postnatal human dental pulp stem cells (DPSCs) in vitro and in vivo. Proc. Natl. Acad. Sci. USA.

[165] Gronthos S., Brahim J., Li W., Fisher L.W., Cherman N., Boyde A., DenBesten P., Robey P.G., Shi S. (2002). Stem cell properties of human dental pulp stem cells. J. Dent. Res..

[166] Sharpe P.T. (2001). Neural crest and tooth morphogenesis. Adv. Dent. Res..

[167] Sharpe P.T. (2016). Dental mesenchymal stem cells. Development.

[168] Cooper P.R., Takahashi Y., Graham L.W., Simon S., Imazato S., Smith A.J. (2010). Inflammation-regeneration interplay in the dentine-pulp complex. J. Dent..

[169] Cao Y., Song M., Kim E., Shon W., Chugal N., Bogen G., Lin L., Kim R.H., Park N.H., Kang M.K. (2015). Pulp-dentin Regeneration: Current State and Future Prospects. J. Dent. Res..

[170] Cooper P.R., Chicca I.J., Holder M.J., Milward M.R. (2017). Inflammation and Regeneration in the Dentin-pulp Complex: Net Gain or Net Loss?. J. Endod..

[171] Xie Z., Shen Z., Zhan P., Yang J., Huang Q., Huang S., Chen L., Lin Z. (2021). Functional Dental Pulp Regeneration: Basic Research and Clinical Translation. Int. J. Mol. Sci..

[172] Van Hassel H.J. (2021). Reprint of: Physiology of the Human Dental Pulp. J. Endod..

[173] Humphrey J.D., Schwartz M.A. (2021). Vascular Mechanobiology: Homeostasis, Adaptation, and Disease. Annu. Rev. Biomed. Eng..

[174] Allen B.J., Frye H., Ramanathan R., Caggiano L.R., Tabima D.M., Chesler N.C., Philip J.L. (2023). Biomechanical and Mechanobiological Drivers of the Transition From PostCapillary Pulmonary Hypertension to Combined Pre-/PostCapillary Pulmonary Hypertension. J. Am. Heart Assoc..

[175] Tonder K.J. (1983). Vascular reactions in the dental pulp during inflammation. Acta Odontol. Scand..

[176] Felaco M., Di Maio F.D., De Fazio P., D’Arcangelo C., De Lutiis M.A., Varvara G., Grilli A., Barbacane R.C., Reale M., Conti P. (2000). Localization of the e-NOS enzyme in endothelial cells and odontoblasts of healthy human dental pulp. Life Sci..

[177] Di Nardo Di Maio F., Lohinai Z., D’Arcangelo C., De Fazio P.E., Speranza L., De Lutiis M.A., Patruno A., Grilli A., Felaco M. (2004). Nitric oxide synthase in healthy and inflamed human dental pulp. J. Dent. Res..

[178] Rafea R., Siragusa M., Fleming I. (2025). The Ever-Expanding Influence of the Endothelial Nitric Oxide Synthase. Basic Clin. Pharmacol. Toxicol..

[179] Shay-Salit A., Shushy M., Wolfovitz E., Yahav H., Breviario F., Dejana E., Resnick N. (2002). VEGF receptor 2 and the adherens junction as a mechanical transducer in vascular endothelial cells. Proc. Natl. Acad. Sci. USA.

[180] Sieminski A.L., Hebbel R.P., Gooch K.J. (2004). The relative magnitudes of endothelial force generation and matrix stiffness modulate capillary morphogenesis in vitro. Exp. Cell Res..

[181] Korkmaz Y., Plomann M., Puladi B., Demirbas A., Bloch W., Deschner J. (2023). Dental Pulp Inflammation Initiates the Occurrence of Mast Cells Expressing the α(1) and β(1) Subunits of Soluble Guanylyl Cyclase. Int. J. Mol. Sci..

[182] Kietadisorn R., Juni R.P., Moens A.L. (2012). Tackling endothelial dysfunction by modulating NOS uncoupling: New insights into its pathogenesis and therapeutic possibilities. Am. J. Physiol. Endocrinol. Metab..

[183] Sies H., Jones D.P. (2020). Reactive oxygen species (ROS) as pleiotropic physiological signalling agents. Nat. Rev. Mol. Cell Biol..

[184] Sies H., Belousov V.V., Chandel N.S., Davies M.J., Jones D.P., Mann G.E., Murphy M.P., Yamamoto M., Winterbourn C. (2022). Defining roles of specific reactive oxygen species (ROS) in cell biology and physiology. Nat. Rev. Mol. Cell Biol..

[185] Münzel T., Sinning C., Post F., Warnholtz A., Schulz E. (2008). Pathophysiology, diagnosis and prognostic implications of endothelial dysfunction. Ann. Med..

[186] Griendling K.K., Touyz R.M., Zweier J.L., Dikalov S., Chilian W., Chen Y.R., Harrison D.G., Bhatnagar A. (2016). Measurement of Reactive Oxygen Species, Reactive Nitrogen Species, and Redox-Dependent Signaling in the Cardiovascular System: A Scientific Statement From the American Heart Association. Circ. Res..

[187] Kuzkaya N., Weissmann N., Harrison D.G., Dikalov S. (2003). Interactions of peroxynitrite, tetrahydrobiopterin, ascorbic acid, and thiols: Implications for uncoupling endothelial nitric-oxide synthase. J. Biol. Chem..

[188] Zou M.H., Shi C., Cohen R.A. (2002). Oxidation of the zinc-thiolate complex and uncoupling of endothelial nitric oxide synthase by peroxynitrite. J. Clin. Investig..

[189] Fleming I., Fisslthaler B., Dimmeler S., Kemp B.E., Busse R. (2001). Phosphorylation of Thr^495^ regulates Ca^2+^/calmodulin-dependent endothelial nitric oxide synthase activity. Circ. Res..

[190] Lin M.I., Fulton D., Babbitt R., Fleming I., Busse R., Pritchard K.A., Sessa W.C. (2003). Phosphorylation of threonine 497 in endothelial nitric-oxide synthase coordinates the coupling of L-arginine metabolism to efficient nitric oxide production. J. Biol. Chem..

[191] Chen C.A., Wang T.Y., Varadharaj S., Reyes L.A., Hemann C., Talukder M.A., Chen Y.R., Druhan L.J., Zweier J.L. (2010). S-glutathionylation uncouples eNOS and regulates its cellular and vascular function. Nature.

[192] Zweier J.L., Chen C.A., Druhan L.J. (2011). S-glutathionylation reshapes our understanding of endothelial nitric oxide synthase uncoupling and nitric oxide/reactive oxygen species-mediated signaling. Antioxid. Redox Signal..

[193] Bode-Böger S.M., Scalera F., Ignarro L.J. (2007). The L-arginine paradox: Importance of the L-arginine/asymmetrical dimethylarginine ratio. Pharmacol. Ther..

[194] Sydow K., Münzel T. (2003). ADMA and oxidative stress. Atheroscler. Suppl..

[195] Böger R.H. (2003). Association of asymmetric dimethylarginine and endothelial dysfunction. Clin. Chem. Lab. Med..

[196] Alp N.J., Channon K.M. (2004). Regulation of endothelial nitric oxide synthase by tetrahydrobiopterin in vascular disease. Arterioscler. Thromb. Vasc. Biol..

[197] Katusic Z.S., d’Uscio L.V. (2004). Tetrahydrobiopterin: Mediator of endothelial protection. Arterioscler. Thromb. Vasc. Biol..

[198] Moens A.L., Kass D.A. (2006). Tetrahydrobiopterin and cardiovascular disease. Arterioscler. Thromb. Vasc. Biol..

[199] Xu J., Wu Y., Song P., Zhang M., Wang S., Zou M.H. (2007). Proteasome-dependent degradation of guanosine 5′-triphosphate cyclohydrolase I causes tetrahydrobiopterin deficiency in diabetes mellitus. Circulation.

[200] Daiber A., Steven S., Vujacic-Mirski K., Kalinovic S., Oelze M., Di Lisa F., Münzel T. (2020). Regulation of Vascular Function and Inflammation via Cross Talk of Reactive Oxygen and Nitrogen Species from Mitochondria or NADPH Oxidase-Implications for Diabetes Progression. Int. J. Mol. Sci..

[201] Landmesser U., Dikalov S., Price S.R., McCann L., Fukai T., Holland S.M., Mitch W.E., Harrison D.G. (2003). Oxidation of tetrahydrobiopterin leads to uncoupling of endothelial cell nitric oxide synthase in hypertension. J. Clin. Investig..

[202] Xu J., Wang S., Wu Y., Song P., Zou M.H. (2009). Tyrosine nitration of PA700 activates the 26S proteasome to induce endothelial dysfunction in mice with angiotensin II-induced hypertension. Hypertension.

[203] Li H., Förstermann U. (2013). Uncoupling of endothelial NO synthase in atherosclerosis and vascular disease. Curr. Opin. Pharmacol..

[204] Chen P.F., Tsai A.L., Wu K.K. (1995). Cysteine 99 of endothelial nitric oxide synthase (NOS-III) is critical for tetrahydrobiopterin-dependent NOS-III stability and activity. Biochem. Biophys. Res. Commun..

[205] Gielis J.F., Lin J.Y., Wingler K., Van Schil P.E., Schmidt H.H., Moens A.L. (2011). Pathogenetic role of eNOS uncoupling in cardiopulmonary disorders. Free Radic. Biol. Med..

[206] Li H., Förstermann U., Xia N., Kuntic M., Münzel T., Daiber A. (2025). Pharmacological targeting of endothelial nitric oxide synthase dysfunction and nitric oxide replacement therapy. Free Radic. Biol. Med..

[207] Chen C.A., De Pascali F., Basye A., Hemann C., Zweier J.L. (2013). Redox modulation of endothelial nitric oxide synthase by glutaredoxin-1 through reversible oxidative post-translational modification. Biochemistry.

[208] Li H., Meininger C.J., Hawker J.R., Haynes T.E., Kepka-Lenhart D., Mistry S.K., Morris S.M., Wu G. (2001). Regulatory role of arginase I and II in nitric oxide, polyamine, and proline syntheses in endothelial cells. Am. J. Physiol. Endocrinol. Metab..

[209] Morris S.M. (2004). Enzymes of arginine metabolism. J. Nutr..

[210] Yang Z., Ming X.F. (2013). Arginase: The emerging therapeutic target for vascular oxidative stress and inflammation. Front. Immunol..

[211] Förstermann U., Xia N., Li H. (2017). Roles of Vascular Oxidative Stress and Nitric Oxide in the Pathogenesis of Atherosclerosis. Circ. Res..

[212] Katusic Z.S. (2007). Mechanisms of endothelial dysfunction induced by aging: Role of arginase I. Circ. Res..

[213] Durante W., Johnson F.K., Johnson R.A. (2007). Arginase: A critical regulator of nitric oxide synthesis and vascular function. Clin. Exp. Pharmacol. Physiol..

[214] Vanhoutte P.M. (2008). Arginine and arginase: Endothelial NO synthase double crossed?. Circ. Res..

[215] Pernow J., Jung C. (2013). Arginase as a potential target in the treatment of cardiovascular disease: Reversal of arginine steal?. Cardiovasc. Res..

[216] Zhao Y., Vanhoutte P.M., Leung S.W. (2015). Vascular nitric oxide: Beyond eNOS. J. Pharmacol. Sci..

[217] Schnabel R., Blankenberg S., Lubos E., Lackner K.J., Rupprecht H.J., Espinola-Klein C., Jachmann N., Post F., Peetz D., Bickel C. (2005). Asymmetric dimethylarginine and the risk of cardiovascular events and death in patients with coronary artery disease: Results from the AtheroGene Study. Circ. Res..

[218] Daiber A., Xia N., Steven S., Oelze M., Hanf A., Kröller-Schön S., Münzel T., Li H. (2019). New Therapeutic Implications of Endothelial Nitric Oxide Synthase (eNOS) Function/Dysfunction in Cardiovascular Disease. Int. J. Mol. Sci..

[219] Schulz E., Wenzel P., Münzel T., Daiber A. (2014). Mitochondrial redox signaling: Interaction of mitochondrial reactive oxygen species with other sources of oxidative stress. Antioxid. Redox Signal..

[220] Stasch J.P., Schmidt P.M., Nedvetsky P.I., Nedvetskaya T.Y., HS S.A., Meurer S., Deile M., Taye A., Knorr A., Lapp H. (2006). Targeting the heme-oxidized nitric oxide receptor for selective vasodilatation of diseased blood vessels. J. Clin. Investig..

[221] Fritz B.G., Hu X., Brailey J.L., Berry R.E., Walker F.A., Montfort W.R. (2011). Oxidation and loss of heme in soluble guanylyl cyclase from Manduca sexta. Biochemistry.

[222] Tawa M., Okamura T. (2022). Factors influencing the soluble guanylate cyclase heme redox state in blood vessels. Vasc. Pharmacol..

[223] Gladwin M.T. (2006). Deconstructing endothelial dysfunction: Soluble guanylyl cyclase oxidation and the NO resistance syndrome. J. Clin. Investig..

[224] Foster M.W., McMahon T.J., Stamler J.S. (2003). S-nitrosylation in health and disease. Trends Mol. Med..

[225] Hess D.T., Matsumoto A., Kim S.O., Marshall H.E., Stamler J.S. (2005). Protein S-nitrosylation: Purview and parameters. Nat. Rev. Mol. Cell Biol..

[226] Sayed N., Baskaran P., Ma X., van den Akker F., Beuve A. (2007). Desensitization of soluble guanylyl cyclase, the NO receptor, by S-nitrosylation. Proc. Natl. Acad. Sci. USA.

[227] Riego J.A., Broniowska K.A., Kettenhofen N.J., Hogg N. (2009). Activation and inhibition of soluble guanylyl cyclase by S-nitrosocysteine: Involvement of amino acid transport system L. Free Radic. Biol. Med..

[228] Sayed N., Kim D.D., Fioramonti X., Iwahashi T., Durán W.N., Beuve A. (2008). Nitroglycerin-induced S-nitrosylation and desensitization of soluble guanylyl cyclase contribute to nitrate tolerance. Circ. Res..

[229] Münzel T., Daiber A., Gori T. (2011). Nitrate therapy: New aspects concerning molecular action and tolerance. Circulation.

[230] Galler K.M., Weber M., Korkmaz Y., Widbiller M., Feuerer M. (2021). Inflammatory Response Mechanisms of the Dentine-Pulp Complex and the Periapical Tissues. Int. J. Mol. Sci..

[231] Kása A., Csortos C., Verin A.D. (2015). Cytoskeletal mechanisms regulating vascular endothelial barrier function in response to acute lung injury. Tissue Barriers.

[232] Dolmatova E.V., Wang K., Mandavilli R., Griendling K.K. (2021). The effects of sepsis on endothelium and clinical implications. Cardiovasc. Res..

[233] Goldberg M., Farges J.C., Lacerda-Pinheiro S., Six N., Jegat N., Decup F., Septier D., Carrouel F., Durand S., Chaussain-Miller C. (2008). Inflammatory and immunological aspects of dental pulp repair. Pharmacol. Res..

[234] Korkmaz Y., Lang H., Beikler T., Cho B., Behrends S., Bloch W., Addicks K., Raab W.H. (2011). Irreversible inflammation is associated with decreased levels of the α1-, β1-, and α2-subunits of sGC in human odontoblasts. J. Dent. Res..

[235] Farges J.C., Alliot-Licht B., Baudouin C., Msika P., Bleicher F., Carrouel F. (2013). Odontoblast control of dental pulp inflammation triggered by cariogenic bacteria. Front. Physiol..

[236] Joffre J., Hellman J., Ince C., Ait-Oufella H. (2020). Endothelial Responses in Sepsis. Am. J. Respir. Crit. Care Med..

[237] Hahn C.L., Falkler W.A., Siegel M.A. (1989). A study of T and B cells in pulpal pathosis. J. Endod..

[238] Izumi T., Kobayashi I., Okamura K., Sakai H. (1995). Immunohistochemical study on the immunocompetent cells of the pulp in human non-carious and carious teeth. Arch. Oral Biol..

[239] Yu C., Abbott P.V. (2007). An overview of the dental pulp: Its functions and responses to injury. Aust. Dent. J..

[240] El Karim I.A., Cooper P.R., About I., Tomson P.L., Lundy F.T., Duncan H.F. (2021). Deciphering Reparative Processes in the Inflamed Dental Pulp. Front. Dent. Med..

[241] Craige S.M., Kant S., Keaney J.F. (2015). Reactive oxygen species in endothelial function-from disease to adaptation. Circ. J..

[242] Sies H. (2015). Oxidative stress: A concept in redox biology and medicine. Redox Biol..

[243] Sies H., Mailloux R.J., Jakob U. (2024). Fundamentals of redox regulation in biology. Nat. Rev. Mol. Cell Biol..

[244] Schulz E., Jansen T., Wenzel P., Daiber A., Münzel T. (2008). Nitric oxide, tetrahydrobiopterin, oxidative stress, and endothelial dysfunction in hypertension. Antioxid. Redox Signal..

[245] Stamler J.S. (1994). Redox signaling: Nitrosylation and related target interactions of nitric oxide. Cell.

[246] Kawanishi H.N., Kawashima N., Suzuki N., Suda H., Takagi M. (2004). Effects of an inducible nitric oxide synthase inhibitor on experimentally induced rat pulpitis. Eur. J. Oral Sci..

[247] Kawashima N., Nakano-Kawanishi H., Suzuki N., Takagi M., Suda H. (2005). Effect of NOS inhibitor on cytokine and COX2 expression in rat pulpitis. J. Dent. Res..

[248] Radi R., Cassina A., Hodara R., Quijano C., Castro L. (2002). Peroxynitrite reactions and formation in mitochondria. Free Radic. Biol. Med..

[249] Frijhoff J., Winyard P.G., Zarkovic N., Davies S.S., Stocker R., Cheng D., Knight A.R., Taylor E.L., Oettrich J., Ruskovska T. (2015). Clinical Relevance of Biomarkers of Oxidative Stress. Antioxid. Redox Signal..

[250] Daiber A., Hahad O., Andreadou I., Steven S., Daub S., Münzel T. (2021). Redox-related biomarkers in human cardiovascular disease–classical footprints and beyond. Redox Biol..

[251] Beckmann J.S., Ye Y.Z., Anderson P.G., Chen J., Accavitti M.A., Tarpey M.M., White C.R. (1994). Extensive nitration of protein tyrosines in human atherosclerosis detected by immunohistochemistry. Biol. Chem. Hoppe Seyler.

[252] Beal M.F., Ferrante R.J., Browne S.E., Matthews R.T., Kowall N.W., Brown R.H. (1997). Increased 3-nitrotyrosine in both sporadic and familial amyotrophic lateral sclerosis. Ann. Neurol..

[253] Zou M.H., Leist M., Ullrich V. (1999). Selective nitration of prostacyclin synthase and defective vasorelaxation in atherosclerotic bovine coronary arteries. Am. J. Pathol..

[254] Strassburger M., Bloch W., Sulyok S., Schüller J., Keist A.F., Schmidt A., Wenk J., Peters T., Wlaschek M., Lenart J. (2005). Heterozygous deficiency of manganese superoxide dismutase results in severe lipid peroxidation and spontaneous apoptosis in murine myocardium in vivo. Free Radic. Biol. Med..

[255] Cai H., Harrison D.G. (2000). Endothelial dysfunction in cardiovascular diseases: The role of oxidant stress. Circ. Res..

[256] Forrester S.J., Kikuchi D.S., Hernandes M.S., Xu Q., Griendling K.K. (2018). Reactive Oxygen Species in Metabolic and Inflammatory Signaling. Circ. Res..

[257] Huang P.L. (2009). eNOS, metabolic syndrome and cardiovascular disease. Trends Endocrinol. Metab..

[258] Hühmer A.F., Gerber N.C., de Montellano P.R., Schöneich C. (1996). Peroxynitrite reduction of calmodulin stimulation of neuronal nitric oxide synthase. Chem. Res. Toxicol..

[259] Zippel N., Loot A.E., Stingl H., Randriamboavonjy V., Fleming I., Fisslthaler B. (2018). Endothelial AMP-Activated Kinase α1 Phosphorylates eNOS on Thr495 and Decreases Endothelial NO Formation. Int. J. Mol. Sci..

[260] Kim S., Dörscher-Kim J. (1989). Hemodynamic regulation of the dental pulp in a low compliance environment. J. Endod..

[261] Wang Z., Zhang J., Li B., Gao X., Liu Y., Mao W., Chen S.L. (2014). Resveratrol ameliorates low shear stress-induced oxidative stress by suppressing ERK/eNOS-Thr495 in endothelial cells. Mol. Med. Rep..

[262] Kong X., Qu X., Li B., Wang Z., Chao Y., Jiang X., Wu W., Chen S.L. (2017). Modulation of low shear stress-induced eNOS multi-site phosphorylation and nitric oxide production via protein kinase and ERK1/2 signaling. Mol. Med. Rep..

[263] Li B., Zhang J., Wang Z., Chen S. (2016). Ivabradine Prevents Low Shear Stress Induced Endothelial Inflammation and Oxidative Stress via mTOR/eNOS Pathway. PLoS ONE.

[264] Chao Y., Ye P., Zhu L., Kong X., Qu X., Zhang J., Luo J., Yang H., Chen S. (2018). Low shear stress induces endothelial reactive oxygen species via the AT1R/eNOS/NO pathway. J. Cell Physiol..

[265] Heitzer T., Brockhoff C., Mayer B., Warnholtz A., Mollnau H., Henne S., Meinertz T., Münzel T. (2000). Tetrahydrobiopterin improves endothelium-dependent vasodilation in chronic smokers: Evidence for a dysfunctional nitric oxide synthase. Circ. Res..

[266] Milstien S., Katusic Z. (1999). Oxidation of tetrahydrobiopterin by peroxynitrite: Implications for vascular endothelial function. Biochem. Biophys. Res. Commun..

[267] Sharina I.G., Martin E. (2017). The Role of Reactive Oxygen and Nitrogen Species in the Expression and Splicing of Nitric Oxide Receptor. Antioxid. Redox Signal..

[268] Weber M., Lauer N., Mülsch A., Kojda G. (2001). The effect of peroxynitrite on the catalytic activity of soluble guanylyl cyclase. Free Radic. Biol. Med..

[269] Tawa M., Shimosato T., Iwasaki H., Imamura T., Okamura T. (2014). Effects of peroxynitrite on relaxation through the NO/sGC/cGMP pathway in isolated rat iliac arteries. J. Vasc. Res..

[270] Melichar V.O., Behr-Roussel D., Zabel U., Uttenthal L.O., Rodrigo J., Rupin A., Verbeuren T.J., Kumar H.S.A., Schmidt H.H. (2004). Reduced cGMP signaling associated with neointimal proliferation and vascular dysfunction in late-stage atherosclerosis. Proc. Natl. Acad. Sci. USA.

[271] Korkmaz Y., Bloch W., Steinritz D., Baumann M.A., Addicks K., Schneider K., Raab W.H. (2006). Bradykinin mediates phosphorylation of eNOS in odontoblasts. J. Dent. Res..

[272] Albanese I., Khan K., Barratt B., Al-Kindi H., Schwertani A. (2018). Atherosclerotic Calcification: Wnt Is the Hint. J. Am. Heart Assoc..

[273] Sawa Y., Yoshida S., Shibata K.I., Suzuki M., Mukaida A. (1998). Vascular endothelium of human dental pulp expresses diverse adhesion molecules for leukocyte emigration. Tissue Cell.

[274] Smith A.J. (2003). Vitality of the dentin-pulp complex in health and disease: Growth factors as key mediators. J. Dent. Educ..

[275] Duncan H.F. (2022). Present status and future directions-Vital pulp treatment and pulp preservation strategies. Int. Endod. J..

[276] Goldberg M., Kulkarni A.B., Young M., Boskey A. (2011). Dentin: Structure, composition and mineralization. Front. Biosci..

[277] Linde A. (1989). Dentin matrix proteins: Composition and possible functions in calcification. Anat. Rec..

[278] Smith A.J., Cassidy N., Perry H., Bègue-Kirn C., Ruch J.V., Lesot H. (1995). Reactionary dentinogenesis. Int. J. Dev. Biol..

[279] Murray P.E., About I., Lumley P.J., Smith G., Franquin J.C., Smith A.J. (2000). Postoperative pulpal and repair responses. J. Am. Dent. Assoc..

[280] Neves V.C.M., Sharpe P.T. (2018). Regulation of Reactionary Dentine Formation. J. Dent. Res..

[281] Smith J.G., Smith A.J., Shelton R.M., Cooper P.R. (2012). Recruitment of dental pulp cells by dentine and pulp extracellular matrix components. Exp. Cell Res..

[282] Yianni V., Sharpe P.T. (2019). Perivascular-Derived Mesenchymal Stem Cells. J. Dent. Res..

[283] Vongsavan N., Matthews B. (1992). The vascularity of dental pulp in cats. J. Dent. Res..

[284] Meyer M.W. (1993). Pulpal blood flow: Use of radio-labelled microspheres. Int. Endod. J..

[285] Takahashi K., Kishi Y., Kim S. (1982). A scanning electron microscope study of the blood vessels of dog pulp using corrosion resin casts. J. Endod..

[286] Kishi Y., Shimozato N., Takahashi K. (1989). Vascular architecture of cat pulp using corrosive resin cast under scanning electron, microscopy. J. Endod..

[287] Corpron R.E., Avery J.K., Lee S.D. (1973). Ultrastructure of capillaries in the odontoblastic layer. J. Dent. Res..

[288] Torabinejad M., Peters D.L., Peckham N., Rentchler L.R., Richardson J. (1993). Electron microscopic changes in human pulps after intraligamental injection. Oral Surg. Oral Med. Oral Pathol..

[289] Münzel T., Daiber A., Gori T. (2013). More answers to the still unresolved question of nitrate tolerance. Eur. Heart J..

[290] Münzel T., Steven S., Daiber A. (2014). Organic nitrates: Update on mechanisms underlying vasodilation, tolerance and endothelial dysfunction. Vasc. Pharmacol..

[291] Ushio-Fukai M., Ash D., Nagarkoti S., Belin de Chantemèle E.J., Fulton D.J.R., Fukai T. (2021). Interplay Between Reactive Oxygen/Reactive Nitrogen Species and Metabolism in Vascular Biology and Disease. Antioxid. Redox Signal..

[292] Szabó C., Ischiropoulos H., Radi R. (2007). Peroxynitrite: Biochemistry, pathophysiology and development of therapeutics. Nat. Rev. Drug Discov..

[293] Pacher P., Beckman J.S., Liaudet L. (2007). Nitric oxide and peroxynitrite in health and disease. Physiol. Rev..

[294] Schulz E., Tsilimingas N., Rinze R., Reiter B., Wendt M., Oelze M., Woelken-Weckmüller S., Walter U., Reichenspurner H., Meinertz T. (2002). Functional and biochemical analysis of endothelial (dys)function and NO/cGMP signaling in human blood vessels with and without nitroglycerin pretreatment. Circulation.

[295] Dulce R.A., Schulman I.H., Hare J.M. (2011). S-glutathionylation: A redox-sensitive switch participating in nitroso-redox balance. Circ. Res..

[296] Landmesser U., Drexler H. (2005). The clinical significance of endothelial dysfunction. Curr. Opin. Cardiol..

[297] Münzel T., Daiber A. (2023). Vascular Redox Signaling, Endothelial Nitric Oxide Synthase Uncoupling, and Endothelial Dysfunction in the Setting of Transportation Noise Exposure or Chronic Treatment with Organic Nitrates. Antioxid. Redox Signal..

[298] Münzel T., Gori T., Bruno R.M., Taddei S. (2010). Is oxidative stress a therapeutic target in cardiovascular disease?. Eur. Heart J..

[299] Cunnington C., Van Assche T., Shirodaria C., Kylintireas I., Lindsay A.C., Lee J.M., Antoniades C., Margaritis M., Lee R., Cerrato R. (2012). Systemic and vascular oxidation limits the efficacy of oral tetrahydrobiopterin treatment in patients with coronary artery disease. Circulation.

[300] McNeill E., Channon K.M. (2012). The role of tetrahydrobiopterin in inflammation and cardiovascular disease. Thromb. Haemost..

[301] Tsai E.J., Kass D.A. (2009). Cyclic GMP signaling in cardiovascular pathophysiology and therapeutics. Pharmacol. Ther..

[302] Sandner P., Follmann M., Becker-Pelster E., Hahn M.G., Meier C., Freitas C., Roessig L., Stasch J.P. (2024). Soluble GC stimulators and activators: Past, present and future. Br. J. Pharmacol..

[303] Sharina I., Martin E. (2023). Cellular Factors That Shape the Activity or Function of Nitric Oxide-Stimulated Soluble Guanylyl Cyclase. Cells.

[304] Friebe A., Englert N. (2022). NO-sensitive guanylyl cyclase in the lung. Br. J. Pharmacol..

[305] Englert N., Burkard P., Aue A., Rosenwald A., Nieswandt B., Friebe A. (2023). Anti-Fibrotic and Anti-Inflammatory Role of NO-Sensitive Guanylyl Cyclase in Murine Lung. Int. J. Mol. Sci..

[306] Follmann M., Griebenow N., Hahn M.G., Hartung I., Mais F.J., Mittendorf J., Schäfer M., Schirok H., Stasch J.P., Stoll F. (2013). The chemistry and biology of soluble guanylate cyclase stimulators and activators. Angew. Chem. Int. Ed..

[307] Stasch J.P., Becker E.M., Alonso-Alija C., Apeler H., Dembowsky K., Feurer A., Gerzer R., Minuth T., Perzborn E., Pleiss U. (2001). NO-independent regulatory site on soluble guanylate cyclase. Nature.

[308] Vakalopoulos A., Wunder F., Hartung I.V., Redlich G., Jautelat R., Buchgraber P., Hassfeld J., Gromov A.V., Lindner N., Bierer D. (2023). New Generation of sGC Stimulators: Discovery of Imidazo[1,2-a]pyridine Carboxamide BAY 1165747 (BAY-747), a Long-Acting Soluble Guanylate Cyclase Stimulator for the Treatment of Resistant Hypertension. J Med. Chem..

[309] Benza R.L., Grünig E., Sandner P., Stasch J.P., Simonneau G. (2024). The nitric oxide-soluble guanylate cyclase-cGMP pathway in pulmonary hypertension: From PDE5 to soluble guanylate cyclase. Eur. Respir. Rev..

[310] Omori K., Kotera J. (2007). Overview of PDEs and their regulation. Circ. Res..

[311] Maurice D.H., Ke H., Ahmad F., Wang Y., Chung J., Manganiello V.C. (2014). Advances in targeting cyclic nucleotide phosphodiesterases. Nat. Rev. Drug Discov..

[312] Samidurai A., Xi L., Das A., Kukreja R.C. (2023). Beyond Erectile Dysfunction: cGMP-Specific Phosphodiesterase 5 Inhibitors for Other Clinical Disorders. Annu. Rev. Pharmacol. Toxicol..

[313] Kass D.A., Champion H.C., Beavo J.A. (2007). Phosphodiesterase type 5: Expanding roles in cardiovascular regulation. Circ. Res..

[314] Lteif C., Ataya A., Duarte J.D. (2021). Therapeutic Challenges and Emerging Treatment Targets for Pulmonary Hypertension in Left Heart Disease. J. Am. Heart Assoc..

[315] Galiè N., Humbert M., Vachiery J.L., Gibbs S., Lang I., Torbicki A., Simonneau G., Peacock A., Vonk Noordegraaf A., Beghetti M. (2016). 2015 ESC/ERS Guidelines for the diagnosis and treatment of pulmonary hypertension: The Joint Task Force for the Diagnosis and Treatment of Pulmonary Hypertension of the European Society of Cardiology (ESC) and the European Respiratory Society (ERS): Endorsed by: Association for European Paediatric and Congenital Cardiology (AEPC), International Society for Heart and Lung Transplantation (ISHLT). Eur. Heart J..

